# Gross motor developmental delay and associated factors among under-five children attending public health facilities of Dessie city, Ethiopia

**DOI:** 10.1186/s12887-023-04461-9

**Published:** 2023-12-18

**Authors:** Kefale Mitiku, Tilaye Nega, Mastewal Arefaynie, Degalem Tilahun, Bereket Kefale, Yitayish Damtie, Bezawit Adane, Melaku Yalew

**Affiliations:** 1 Department of Physiology, College of Medicine and Health Sciences, Injibara University, Injibara, Ethiopia; 2 Department of Developmental Psychology, College Social Sciences, Wollo University, Dessie, Ethiopia; 3 Department of Reproductive and Family Health, School of Public Health, College of Medicine and Health Sciences, Wollo University, Dessie, Ethiopia; 4 Department of Pediatrics, School of Medicine, College of Medicine and Health Sciences, Gondar University, Gondar, Ethiopia; 5 Department of Reproductive Health, School of Public Health, College of Medicine and Health Sciences, Injibara University, Injibara, Ethiopia; 6 Department of Epidemiology and Biostatistics, School of Public Health, College of Medicine and Health Sciences, Injibara University, Injibara, Ethiopia

**Keywords:** Gross motor, Developmental delay, Children, Dessie, Ethiopia

## Abstract

**Background:**

Child psychomotor development and factors affecting it today is the subject of interest of many studies, in particular by the experts involved in the protection and improvement of children’s health. There is limited evidence on developmental delay among under-five children in low-income countries like Ethiopia. The aim of this study was to assess gross motor developmental delay and associated factors among under-five children attending public health facilities of Dessie city, Ethiopia.

**Methods:**

Facility based cross sectional study design was used among under-five children attending under-five OPD in public health facilities of Dessie town from July 1, 2020 to August 15, 2021. A total of, 417 under-five children were systematically selected based on their average number of clients in a month. A pretested structured questionnaire was used for data collection, and data was entered into Epi-data 3.1 version and it was exported to STATA version 14 for analysis. Binary logistic regression analysis was used to identify factors associated with the outcome variable. Odds ratio with 95% confidence interval was used to show the strength and direction of association respectively and *P*-value less than 0.05 is used to declare statistical significance.

**Results:**

The overall proportion of gross motor developmental delay among under-five children attending health facilities of Dessie city, Ethiopia was 16.31%, 95% CI: (13.05, 20.19). Increased age of the child [AOR = 0.97, 95% CI: (0.96, 0.99)], increased gestational age during pregnancy [AOR = 0.47, 95% CI: (0.37, 0.65)], being male [AOR = 5.26, 95% CI: (1.76, 15.67)], having history of alcohol intake during pregnancy [AOR = 7.40, 95% CI: (2.36, 23.25)], taking iron during pregnancy [AOR = 0.04, 95% CI: (0.01, 0.15)], facing fetal and/or maternal complication [AOR = 4.98, 95% CI: (1.20, 20.62)], having instrumental delivery [AOR = 9.78, 95% CI: (2.48, 38.60)] were significantly associated with gross motor developmental delay.

**Conclusions:**

The gross motor developmental delay among under-five children was higher as compared to other literatures. This study indicated that, age and sex of the child, iron and alcohol intake during pregnancy, gestational age, mode of delivery and any complication to her and or her neonate were independent variables which showed statistical significant association. The physicians should advise mothers to take iron-folic acid supplement properly and to avoid intake of alcohol during pregnancy. In addition, they should focus on those mothers who faced any complication to her and/or her neonate and better to discourage instrumental delivery unless there are no other options.

**Supplementary Information:**

The online version contains supplementary material available at 10.1186/s12887-023-04461-9.

## Background

Child development can be defined as the process that begins in intrauterine life and involves physical growth, neurological maturation and the construction of behavior-related skills [1]. A process of change in which the child learns to master more complex levels of movement, thinking, feelings and relationships with others also can be taken as child development. It occurs when the child interacts with people, things, and other stimuli in their biophysical and social environment and learns from them [2]. The non-standardized definition also viewed it as a condition in which the child does not reach skills in accordance with the sequence of predetermined stages [3].

Based on theoretical perspectives, delays in the development of motor skills are interpreted from three different approaches, although they are not mutually exclusive. The comparative deficit approach, the social interaction approach, and the adaptive compensation approach [4].

Infant mortality, morbidity, prevalence of disability, living conditions and education of children, especially the under-fives are some of developmental indices which determine a country’s future human resource development. At early child development, an outcome of the survival and care practices adopted in a particular setting, is objectively reflected in the developmental status of children, any delay, dissociation or deviation in the development of children and its causes/contributory factors may be indicative of the need for strengthening the existing programs or the need for exploring and initiating new possibilities [5].

Worldwide, it is estimated that 200 million children younger than five years of age are at risk of not reaching their full development. Despite the prevalence of developmental delay is not certainly known, World Health Organization (WHO) indicate that 10% of the population of any country consists of individuals with some type of disability, with a rate of 4.5% among those younger than five years of age [3]. Psychomotor development and/or language disorders in children under the age of 6 years is estimated as 12–16% [6]. The United State of America survey revealed that, about 16% of children are affected by various disabilities caused by speech and language delay, mental retardation, learning disabilities and emotional/behavioral problems, however only 30% of such children were identified before school entrance age [7]. A preliminary assessment revealed that the prevalence of a psychomotor delay as determined by the global development quotient of children living in Mygoma orphanage was 25% in children under 16 months, and 19% in those age 16 to 31 months [8].

In the developed countries, there is a general consensus regarding the importance of monitoring children’s development through systematic screening. Developmental screening is a globally adopted measure by which children at various set ages (2 to 60 months) are routinely assessed to detect those at high risk for significant unsuspected deviation from the normal [7]. The necessity to screen a child’s development at a very young age is obvious since research has shown that the earlier the intervention the better the developmental outcome. Consequently, professional pediatric societies recommend the identification of those children with a developmental delay before the age of 6 years [6]. A number of studies have shown that early intervention programs are not only cost effective but they are also lifelong benefit and optimal developmental attainment. The earlier the intervention the greater the benefit will be [9]. In the first few years of life, growth and development is an important health indicator of children. Mortality and morbidity among children under the age of five years has strong associations with severe growth retardation [1], while impaired psycho-social and intellectual development and learning ability is strongly associated with developmental delay [10].

Gross motor developmental delays in early childhood are often associated different factors. For instance, child factors (age, sex, no of sibling, birth weight and nutritional status) [2, 11–13], paternal factors (age of mother, marital status, mother education, father education, place of residence and ethnicity) [14, 15], obstetric factors (gestational age, birth spacing, mode of delivery, complication during labor and or delivery) [6, 16, 17] and illness and behavioral factors (childhood exposure during child hood, childhood exposure to malaria, type of salt they used, mother take iron, r/ship b/n mother and child) [4, 18, 19].

Even though Ethiopia tried a lot to prevent child developmental delay by providing safety net, fortification of iodine in salt and the like and most of which would have been either preventable or manageable if it can detected early [7], it is still overlooked in determining its magnitude and associated factors. Since there is no similar study conducted in the study area and Ethiopia is diversified country and the problem varied, therefore, it will generate relevant information that will fill this gap. The aim of this study was to assess gross motor developmental delay and associated factors among under-five children attending public health facilities of Dessie city, Ethiopia.

## Method and materials

### Study area, period, design and population

This study was conducted in public health facilities of Dessie town, Amhara Regional State, Ethiopia from June 1, 2020 to August 15, 2021. Dessie is the central town of South Wollo Zone, which is located 401 km to the North of Addis Ababa. According to the town administrative health office report the town had 10 urban and 6 rural kebeles and the estimated population size was 218,471 of which, 102,378 (46.86%) are males and 116,093 (53.14%) are females. The town is found at an altitude of 2470 to 2550 m above sea level and the temperature ranges from 15 to 17 oc. There are five hospitals and eight health centers and twenty seven private clinics, of which 10 are public health institutions.

Health facility based cross sectional study design was conducted. All under-five children attending under-five OPD in selected health facilities were the source population. All under-five children attending under-five OPD in selected health facilities and available during the study period was the study population.

### Sample size determination and procedure

The sample size was determined by single population proportion formula by considering the following assumptions. Proportion of gross motor developmental delay in Ethiopia is 50%, since there is no any previous study, 95% of confidence level and allowed margin of error 5%.

$$n = \frac{{{{\left( {Z\alpha /2} \right)}^2}p{{\left( {1 - p} \right)}^{}}}}{{{d^2}}}$$

$$n = \frac{{{{1.96}^2}X0.5(1 - 0.5)}}{{{{(0.05)}^2}}} = 385$$

After adding 10% non-response rates, the final sample size for this study is 424.

**Fig. 1:**
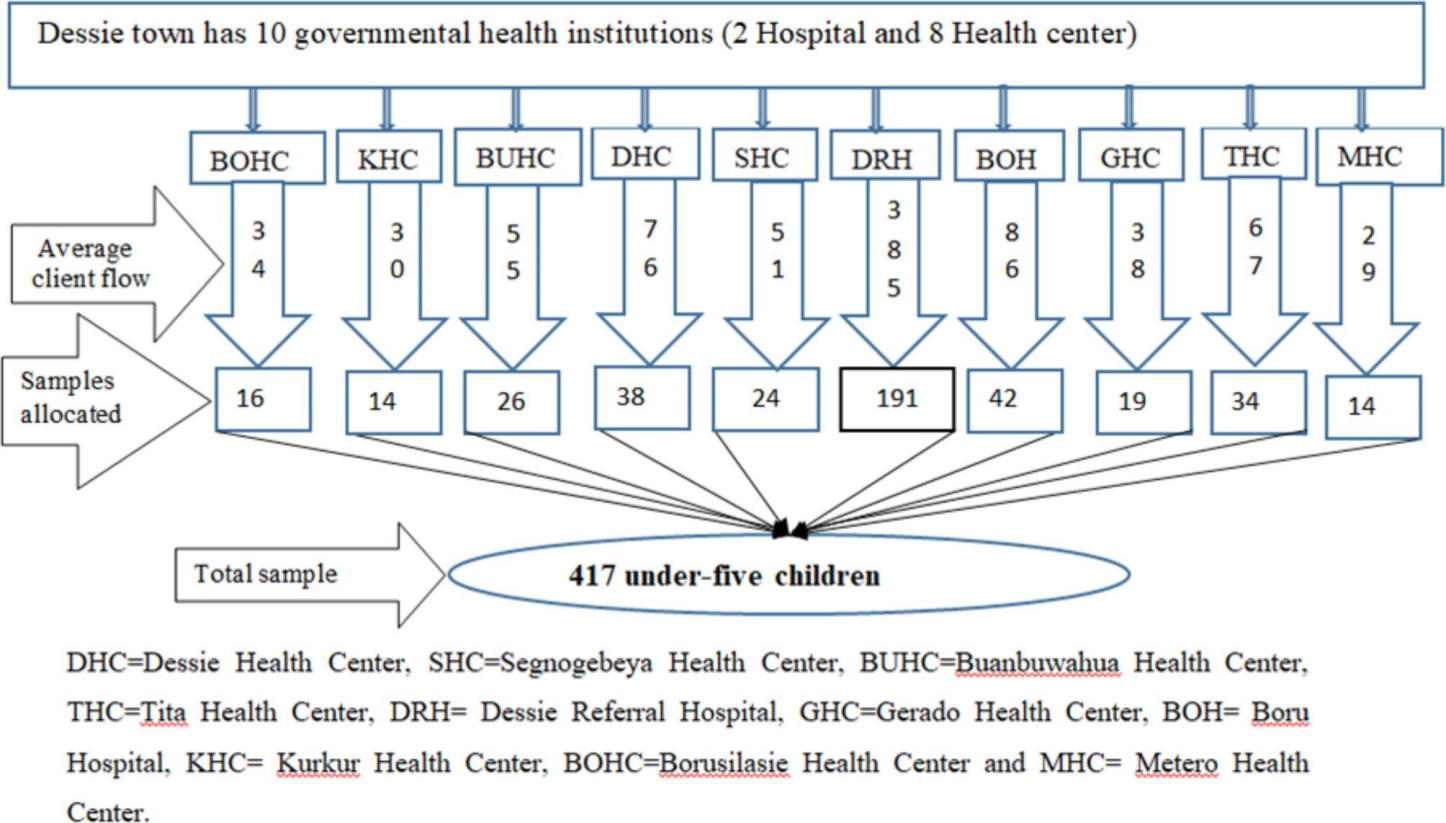
Schematic diagram of sampling procedure for gross motor developmental delay and associated factors among under-five children attending under-five OPD in public health facilities of Dessie city, Ethiopia

The calculated sample size was proportionately allocated based on the average number of client flow per month to each health facility and study participants were selected by systematically until reaching the final sample size (Fig. 1).

### Outcome measurement

The care givers were interviewed and the outcome variable was measured based on Child Developmental Inventory (CDI) which consists 28 item checklists composed of yes/no statements about the child’s development. Each scale is scored by tallying the “yes” answers; a child who receives a score that is 2 SDs below the mean is graded as delayed otherwise normal [20, 21].

### Data collection tools procedures and quality control

Data were collected through interviewer administered questionnaire that were developed from previous done similar literatures [1, 20]. The caregivers were asked for their valuable information after they have finished their primary intention to visit health facility in separate room and the data were filled in the questionnaire. Some of the questionnaires were also filled by observing child individual folder/card and height and weight of the child was also recorded. Two BSc public health officer supervisors and 10 BSc nurses who were working in another health facility were employed as data collectors.

The questionnaire was developed in English language and translated to Amharic again back translated to English to check its consistency by language experts. Supervisors and data collectors were trained on the objective of the study, how to approach participants, measure height and weight and take informed consent. Before entering to the actual data collection, the tool was pre-tested on 5% of the sample in Kombolcha health center and necessary modification was done according to the result of pretest. The data was checked by supervisors and principal investigators daily.

### Data processing and analysis

After collecting the data, it was checked, coded, cleaned and entered in STATA version 14. The results were presented using texts, frequency, proportion, graphs and other summary measures were also computed to describe the study population. Binary logistic regression model was used to identify association between each independent variable and outcome variable and statistical significance was determined using odds ratios with the corresponding *P*-value.

Multi-colinearity between independent variables was checked using variance inflation factor as well as standard error and Hosmer- Lemeshow test was used to check model fitness. In the final model those variables with *p*-value less than 0.05 was considered as statistical significant and it was presented on odds ratio (OR), with 95% confidence interval (CI) to show the strength and direction of association respectively.

## Results

### Socio-demographic characteristics of the respondents

Four hundred seventeen children with their caregivers were participated in this study and the response rate was 98.35%. The median (IQR) age of the child and the caregiver/mother was 30 (± 29) months and 30 (± 8) years respectively. Two hundred twenty two (53.24%) caregiver/mothers were educated to secondary and above whereas 100 (23.98%) were not formally educated. About one hundred fifty-nine (38.13%) children were females. In terms of ethnicity and occupation, 396 (94.96%) were Amhara and 262 (62.83%) mothers were house-wife (Table 1).

**Table 1:** Socio-demographic characteristics of the children and parents who attended in health facilities of Dessie city, Ethiopia

Variables	Category	Frequency	Percentage
Sex of the child	Male	258	61.99
	Female	159	38.01
Place of residence	Rural	119	28.54
	Urban	298	71.46
Marital status	Married	386	92.57
	Divorced	24	5.75
	Widowed	7	1.68
Educational status of father	Can’t read and write	44	10.55
	Can read and write only	24	5.76
	Grade 1–8	106	25.42
	Grade 9–12	97	23.26
	College and above	146	35.01
Educational status of mother	Can’t read and write	89	21.34
	Can read and write only	11	2.64
	Grade 1–8	95	22.78
	Grade 9–12	87	20.86
	College and above	135	32.37
Ethnicity	Amhara	396	94.96
	Oromo	13	3.12
	Others**	8	1.92
Occupation	Government employee	92	22.06
	Merchant	50	11.99
	Private employee	8	1.92
	House wife	262	62.83
	Others***	5	1.20

Note: *- Protestant and Catholic, **- Tigrie and Afar and ***- student and job less

### Obstetric, illness and behavioral factors

Out of all study participants, 262 (62.83%) children were delivered vaginal spontaneously. Two hundred eighty-seven (68.82%) mothers had history of antepartum and/or postpartum complication to the mother and/or the child. One hundred sixteen (23.60%) and two hundred thirty-four (56.12%) mothers didn’t take iron and take alcohol during their pregnancy respectively. Forty-one (10.33%) of the children had history of malaria exposure during pregnancy. The mean (SD) duration of pregnancy was 38.9 (1.39) weeks. The mean (SD) number of siblings was 1.35 (1.55) (Table 2).

**Table 2:** Obstetric, illness and behavioral related data of mothers attending health facilities of Dessie city, Ethiopia

Variables	Category	Frequency	Percentage
Alcohol intake during pregnancy	Yes	234	56.12
	No	183	43.88
Any maternal/fetal complication	Yes	287	68.82
	No	130	31.18
Iron intake during pregnancy	Yes	301	76.40
	No	116	23.60
Malaria exposure during pregnancy	Yes	41	9.83
	No	376	91.17
Iodized salt used	Yes	325	81.86
	No	72	18.14
Mode of delivery	SVD	262	62.83
	Instrumental	71	17.03
	C/S	84	20.14
Family history of developmental delay	Yes	34	8.15
	No	383	91.85
Infection in the past two weeks	Yes	114	29.16
	No	277	70.84

SVD-Spontaneous vaginal delivery and C/S- Cesarean section

### Proportion of gross motor developmental delay

**Fig. 2:**
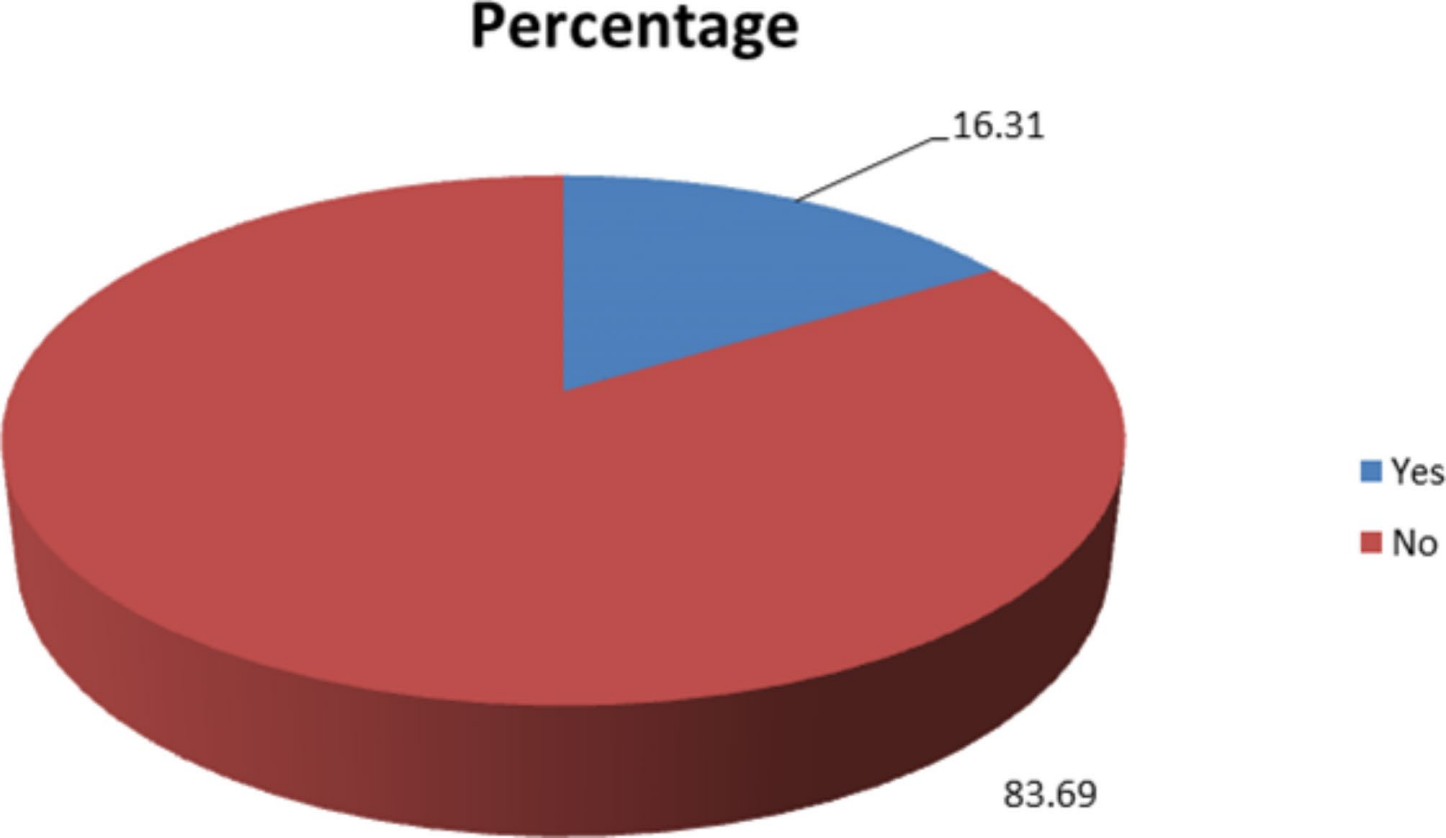
The overall proportion of gross motor developmental delay among under-five children attending health facilities of Dessie city, Ethiopia

The overall proportion of gross motor developmental delay among under-five children attending health facilities of Dessie city, Ethiopia was demonstrated in figure below and its 95% CI: (13.05, 20.19) (Fig. 2).

### Factors associated gross motor developmental delay

In Bivariable logistic regression, eleven variables (age of the child, gestational age, sex of the child, educational status of the mother, educational status of the father, mode of delivery, alcohol intake during pregnancy, iron intake during pregnancy, type of household salt used, family history of GMDD, and had history of complication to her and/or her child during pregnancy) with *P*-value less than 0.2 were considered in multi variable analysis. Multivariable analysis result showed that age of the child, gestational age, sex of the child, mode of delivery, alcohol intake during pregnancy, iron intake during pregnancy, and any fetal and/or maternal complication were found to have significant statistical association with GMDD.

As age of the child increased in one month, the probability of log of odds of gross motor developmental delay decreased by 4% [AOR = 0.97, 95% CI: (0.96, 0.99)]. Similarly, as the gestational age during pregnancy increased in one month, the probability of log of odds of gross motor developmental delay decreased by 66% [AOR = 0.47, 95% CI: (0.37, 0.65)].

The odds of gross motor developmental delay among males were five times more likely than females [AOR = 5.26, 95% CI: (1.76, 15.67)]. Those under-five children whose mothers took alcohol while she was pregnant were seven times more likely to develop gross motor developmental delay as compared to not [AOR = 7.40, 95% CI: (2.36, 23.25)]. However, those under-five children whose mothers took iron while she was pregnant were 99% less likely to develop gross motor developmental delay as compared to not [AOR = 0.04, 95% CI: (0.01, 0.15)].

Those under-five children whose mothers faced fetal and/or maternal complication while she gave birth were five times more likely to develop gross motor developmental delay as compared to not [AOR = 4.98, 95% CI: (1.20, 20.62)]. Lastly, the odds of gross motor developmental delay among children who were delivered through instrument were 10 times more likely than spontaneous vaginal delivery [AOR = 9.78, 95% CI: (2.48, 38.60)] (Table 3).

**Table 3:** Bivariable and multivariable binary logistic regression for gross motor developmental delay among under-five children attending health facilities of Dessie city, Ethiopia

Variables	Category	GMDD		COR	(95% CI)		AOR	95% CI	
		Yes	No		L CI	UCI		L CI	UCI
Age of the child in months				0.97	0.96	0.99	**0.95**	**0.92**	0.99*
Gestational age in weeks				0.47	0.37	0.65	**0.51**	**0.34**	0.78*
Educational status of the mother	Not formally educated	21	79	1	1		1		
	Grade 1–8	25	70	1.34	0.69	2.61	1.45	0.31	6.89
	Grade 9–12	14	73	0.72	0.34	1.52	0.25	0.04	1.61
	College & above	8	127	0.24	0.10	0.56	0.16	0.01	1.84
Educational status of the father	Not formally educated	22	46	7.28	3.13	16.94	4.99	0.65	38.20
	Grade 1–8	11	95	1.76	0.70	4.42	1.14	0.19	6.99
	Grade 9–12	26	71	5.57	2.48	12.54	0.45	0.09	2.39
	College & above	9	137				1		
Mode of delivery	SVD	32	230				1		
	Instrumental	25	46	3.91	2.12	7.20	**9.78**	**2.48**	**38.60**
	C/S	11	73	1.08	0.52	2.26	2.95	0.72	12.03
Iron intake during pregnancy	Yes	33	268	0.28	0.17	0.49	**0.04**	**0.01**	0.15**
	No	35	81				1		
Alcohol intake during pregnancy	Yes	56	178	4.48	2.32	8.66	**7.40**	**2.36**	23.25*
	No	12	171				1		
Type of salt used in the household	Iodized	46	279	0.43	0.23	0.78	0.54	0.15	1.86
	Non-iodized	20	52				1		
Family history of GMDD	Yes	10	24	2.33	1.06	5.14	3.49	0.37	32.96
	No	58	325				1		
Fetal/maternal complication	Yes	40	247	0.59	0.35	1.01	**4.98**	**1.20**	20.62*
	No	28	102				1		
Sex of the child	Male	36	125	2.02	1.19	3.40	**5.26**	**1.76**	15.67*
	Female	32	224				1		

AOR- Adjusted Odds Ratio, COR- Crude Odds Ratio, LCI-lower confidence interval, UCI-upper confidence interval, GMDD- gross motor developmental delay, 1- Reference, *-(*P*-value < 0.05) and **-(*P*-value < 0.01) in multivariable analysis respectively

## Discussion

This facility based cross sectional-study was the first study done on gross motor developmental delay among under-five children in public health facilities of Dessie town. In this study, the proportion of gross motor developmental delay among under-five children was 16.31%. Variables like age and sex of the child, iron and alcohol intake during pregnancy, gestational age, mode of delivery and any complication to her and or her neonate were significantly associated to gross motor developmental delay.

The proportion of gross motor developmental delay among under-five children is comparable with a study conducted in India 19.80% [22]. In addiction, the finding of this study is higher than a study conducted in Ghana 6.7% [7]. In the contrary, it is lower than the studies conducted in Thailand (37.10%) [10] and Mexico (55.10%) [2]. The possible reason for this observed discrepancy may be due to the difference in the tools used to measure gross motor developmental delay and may be due to the difference in children health policy among countries.

As age of the child increased in one month, the probability of log of odds of gross motor developmental delay decreased by 4%. The result of this study is in agreement with a study conducted in France [6]. Nevertheless, the finding of this study is contrary to a study conducted in Indonesia which states that the probability of gross motor developmental delay decreased as the child goes above age two years [9]. The possible reason for this discrepancy may be due to the difference in the context of the country in which the research was undertaken.

Similarly, as the gestational age during pregnancy increased in one month, the probability of log of odds of gross motor developmental delay decreased by 66%. The result of this study is in agreement with a study conducted in Ghana [7]. The possible reason for this may be due to the fact that certain structural and functional cells and organs that have to be completed in uterus play a paramount role in child development.

The odds of gross motor developmental delay among males were five times more likely than females. The finding of this study is consistent with a study conducted in Baghdad [12]. The possible reason for this may be due to the fact that testosterone hormone in males affect development.

Those under-five children whose mothers took alcohol while they were pregnant were seven times more likely to develop gross motor developmental delay as compared to not. However, those under-five children whose mothers took iron while they were pregnant were 99% less likely to develop gross motor developmental delay as compared to not. The possible reason for this may be due to the fact that iron is very important in neurometabolism, myelination and neutransmitters function during brain development.

Those under-five children whose mothers faced fetal and/or maternal complication while they gave birth were five times more likely to develop gross motor developmental delay as compared to not. The finding of this study is consistent with a study conducted in Baghdad [12]. The possible reason for this may be due to the fact that any type of infection she/he faced during pregnancy or postpartum can cross the placenta barrier and it will cause brain cell damage that may predispose the child for gross motor developmental delay.

Lastly, the odds of gross motor developmental delay among children who were delivered through instrument were 10 times more likely than spontaneous vaginal delivery. The possible reason for this may be due to the fact that instrumental delivery may increase the risk of infection and usually cause head injury that may attribute for gross motor developmental delay.

Even if this study is the first in generating and testing hypothesis related to gross motor developmental delay in Ethiopia as strength, it is not without limitation. Firstly, the study was only on governmental health facilities that may not represent experience of private health facilities delivery care. Lastly, the study was conducted in health facility that may not truly represent the real community exposure.

## Conclusions

The gross motor developmental delay among under-five children was higher as compared to other literatures. This study indicated that, age and sex of the child, iron and alcohol intake during pregnancy, gestational age, mode of delivery and any complication to her and or her neonate were independent variables which showed statistical significant association with gross motor developmental delay in the study area. The physicians should advise mothers to take iron-folic acid supplement properly and to avoid intake of alcohol during pregnancy. In addition, they should focus on those mothers who faced any complication to her ad/or her neonate and better to discourage instrumental delivery unless there is no other options. Further researchers better to study it in community based or prospective study design.

### Electronic supplementary material

Below is the link to the electronic supplementary material.

Supplementary Material 1

## Data Availability

The datasets used and/or analyzed during the current study are attached with the manuscript as supporting information (see supplementary file 1).
