# Supplementary material for: Gross motor developmental delay and associated factors among under-five children attending public health facilities of Dessie city, Ethiopia
Source: BMC Pediatr. 2023 Dec 18;23:638. doi: 10.1186/s12887-023-04461-9 (PMC10726544; doi:10.1186/s12887-023-04461-9)
Supplement: Supplementary file 1 — Supplementary Material 1 [file 12887_2023_4461_MOESM1_ESM.pdf]

11	0	3300	1. yes	1	8900	6	21	2. married	4. grade 9-
39	0	3000	1. yes	80	11000	6	20	2. married	3. grade 1-
8	1	3750	1. yes	72	9000	6	32	2. married	5. college ‹
48	2	3000	1. yes	99	14400	6	35	2. married	2. oly read
56	2	3500	1. yes	99	14900	6	29	2. married	5. college ‹
59	1	3000	1. yes	118	18900	6	25	2. married	4. grade 9-
31	0	3000	1. yes	88	11300	6	30	2. married	5. college ‹
18	1	3900	1. yes	3	8700	7	32	2. married	3. grade 1-
25	3	4500	1. yes	89	12500	6	34	2. married	2. oly read
53	0	3700	1. yes	102	14400	6	30	2. married	4. grade 9-
42	3	31000	1. yes	101	14100	6	45	2. married	2. oly read
54	0	3400	1. yes	102	15100	7	28	2. married	5. college ‹
34	0		1. yes	89	11300	6	28	2. married	4. grade 9-
10	0	3500	1. yes	73	9500	6	26	3. divorcec	5. college ‹
8	1	2100	1. yes	71	9900	6	32	2. married	3. grade 1-
42	2	3000	1. yes	98	12000	6	26	2. married	3. grade 1-
39	2		1. yes	88	11000	8	33	4. widowec	3. grade 1-
31	2		1. yes	91	12000	6	32	2. married	4. grade 9-
29	0		1. yes	84	10500	6	25	2. married	5. college ‹
11	1		1. yes	69	7800	7	35	2. married	4. grade 9-
59	3		1. yes	110	17000	6	35		3. grade 1-
36	0	2900	1. yes	100	13100	6	25	2. married	3. grade 1-
12	1	2500	1. yes	76	8500	6	35	2. married	5. college ‹
17	1	3000	1. yes	74	14000	6	28	2. married	3. grade 1-
11	0		1. yes	72	8500	4	24	2. married	4. grade 9-
59	1		1. yes	115	19500	8	35	2. married	2. oly read
19	1	2000	1. yes	77	9500	6	28	2. married	3. grade 1-
59	3		0. no	102	14400	7	35	2. married	2. oly read
18	1	2500	1. yes	81	10000	5	30	2. married	5. college ‹
24	10		1. yes	76	13500	6	23	2. married	3. grade 1-
28	0	3400	1. yes	98	1200	5	29	2. married	5. college ‹
14	0	3000	1. yes	6	9100	6	28	2. married	5. college ‹
14	0	3000	1. yes	80	6	24	24	2. married	5. college ‹
11	0	3500	1. yes	72	9400	6	19	2. married	3. grade 1-
17	1	3000	1. yes	79	8900	6	22	2. married	3. grade 1-
59	0	1800	1. yes	110	5	30	30	2. married	3. grade 1-
48	3		1. yes	98	14600	12	30	2. married	3. grade 1-
42	2	3000	1. yes	98	12000	6	26	2. married	3. grade 1-
32	1	4000	1. yes	95	14100	6	30	2. married	3. grade 1-
7	4	3000	1. yes	74	8400	8	30	2. married	2. oly read
45	5	3000	1. yes	110	16600	3	35	2. married	2. oly read
31	4	3000	1. yes	90	10500	1	36	2. married	2. oly read
43		3300	1. yes	100	14600	6	27	2. married	4. grade 9-
17	3		1. yes	6	9400	7	30	2. married	2. oly read
13	1		1. yes	71	9000	8	30	2. married	2. oly read
39	1		1. yes	73	9000	3	22	2. married	2. oly read
11	0	3500	1. yes	73	8800	6	25	2. married	5. college ‹
50		3900	1. yes	98	15200	6	26	2. married	5. college ‹
59	0	2500	1. yes	106	14200	6	28	2. married	5. college ‹
25	1	3000	1. yes	89	15200	6	35	2. married	5. college ‹
53	1	3500	1. yes	110	16.5	6	35	2. married	3. grade 1-
7	0	3500	1. yes	70	7500	5	25	2. married	5. college ‹

9	1	3500	1. yes	67	7500	5	30	2. married	5. college (
7	2	2800	1. yes	65	8000	6	32	2. married	2. oly read
36	0	3000	1. yes	105	14000	6	24	2. married	4. grade 9-
36	0	3600	1. yes	104	15000	6	28	3. divorcec	5. college (
36	0	3500	1. yes	100	12000	6	26	2. married	5. college (
10	0		1. yes	68	9000	6	22	3. divorcec	2. oly read
34	1	3500	1. yes	97	12500	6	28	2. married	5. college (
59		4000	1. yes	100	15300	24	37	2. married	3. grade 1-
10	0	1600	1. yes	70	8000	6	25	2. married	5. college (
11	0	2400	1. yes	70	8900	6	26	2. married	3. grade 1-
29	0	3200	1. yes	95	12000	6	31	2. married	5. college (
25	1	4500	1. yes	85	12000	6	32	2. married	2. oly read
10	1	3000	1. yes	3	8000	6	28	2. married	4. grade 9-
30	6	3100	1. yes	84	10900	6	35	2. married	2. oly read
20	2	3000	1. yes	84	10500	9	23	2. married	4. grade 9-
30		3000	1. yes	89	12100	6	23	2. married	4. grade 9-
11	0	3300	1. yes	1	8900	6	21	2. married	4. grade 9-
48	2	3000	1. yes	99	14400	6	35	2. married	2. oly read
56	2	3500	1. yes	99	14900	6	29	2. married	5. college (
25	3	4500	1. yes	89	12500	6	34	2. married	2. oly read
53	0	3700	1. yes	102	14400	6	30	2. married	4. grade 9-
42	3	31000	1. yes	101	14100	6	45	2. married	2. oly read
54	0	3400	1. yes	102	15100	7	28	2. married	5. college (
10	0	3500	1. yes	73	9500	6	26	3. divorcec	5. college (
11	1		1. yes	69	7800	7	35	2. married	4. grade 9-
59	3		1. yes	110	17000	6	35		3. grade 1-
17	1	3000	1. yes	74	14000	6	28	2. married	3. grade 1-
18	1	2500	1. yes	81	10000	5	30	2. married	5. college (
28	0	3400	1. yes	98	1200	5	29	2. married	5. college (
14	0	3000	1. yes	6	9100	6	28	2. married	5. college (
11	0	3500	1. yes	72	9400	6	19	2. married	3. grade 1-
43		3300	1. yes	100	14600	6	27	2. married	4. grade 9-
25	1	3000	1. yes	89	15200	6	35	2. married	5. college (
59		4000	1. yes	100	15300	24	37	2. married	3. grade 1-
10	1	3000	1. yes	3	8000	6	28	2. married	4. grade 9-
30	6	3100	1. yes	84	10900	6	35	2. married	2. oly read
8	1	3750	1. yes	72	9000	6	32	2. married	5. college (
56	2	3500	1. yes	99	14900	6	29	2. married	5. college (
8	1	2100	1. yes	71	9900	6	32	2. married	3. grade 1-
39	2		1. yes	88	11000	8	33	4. widowec	3. grade 1-
31	2		1. yes	91	12000	6	32	2. married	4. grade 9-
29	0		1. yes	84	10500	6	25	2. married	5. college (
36	0	2900	1. yes	100	13100	6	25	2. married	3. grade 1-
12	1	2500	1. yes	76	8500	6	35	2. married	5. college (
17	1	3000	1. yes	74	14000	6	28	2. married	3. grade 1-
11	0		1. yes	72	8500	4	24	2. married	4. grade 9-
14	0	3000	1. yes	6	9100	6	28	2. married	5. college (
43		3300	1. yes	100	14600	6	27	2. married	4. grade 9-
17	3		1. yes	6	9400	7	30	2. married	2. oly read
53	1	3500	1. yes	110	16.5	6	35	2. married	3. grade 1-
9	1	3500	1. yes	67	7500	5	30	2. married	5. college (
29	0	3200	1. yes	95	12000	6	31	2. married	5. college (

42	3	31000	1. yes	101	14100	6	45	2. married	2. oly read
54	0	3400	1. yes	102	15100	7	28	2. married	5. college (
10	1	3000	1. yes	3	8000	6	28	2. married	4. grade 9-
30	6	3100	1. yes	84	10900	6	35	2. married	2. oly read
11	0	3300	1. yes	1	8900	6	21	2. married	4. grade 9-
39	0	3000	1. yes	80	11000	6	20	2. married	3. grade 1-
8	1	3750	1. yes	72	9000	6	32	2. married	5. college (
48	2	3000	1. yes	99	14400	6	35	2. married	2. oly read
56	2	3500	1. yes	99	14900	6	29	2. married	5. college (
31	0	3000	1. yes	88	11300	6	30	2. married	5. college (
25	3	4500	1. yes	89	12500	6	34	2. married	2. oly read
53	0	3700	1. yes	102	14400	6	30	2. married	4. grade 9-
54	0	3400	1. yes	102	15100	7	28	2. married	5. college (
10	0	3500	1. yes	73	9500	6	26	3. divorcec	5. college (
8	1	2100	1. yes	71	9900	6	32	2. married	3. grade 1-
42	2	3000	1. yes	98	12000	6	26	2. married	3. grade 1-
39	2		1. yes	88	11000	8	33	4. widowec	3. grade 1-
11	1		1. yes	69	7800	7	35	2. married	4. grade 9-
17	1	3000	1. yes	74	14000	6	28	2. married	3. grade 1-
11	0		1. yes	72	8500	4	24	2. married	4. grade 9-
59	3		0. no	102	14400	7	35	2. married	2. oly read
28	0	3400	1. yes	98	1200	5	29	2. married	5. college (
14	0	3000	1. yes	6	9100	6	28	2. married	5. college (
14	0	3000	1. yes	80	6	24	24	2. married	5. college (
17	1	3000	1. yes	79	8900	6	22	2. married	3. grade 1-
48	3		1. yes	98	14600	12	30	2. married	3. grade 1-
32	1	4000	1. yes	95	14100	6	30	2. married	3. grade 1-
45	5	3000	1. yes	110	16600	3	35	2. married	2. oly read
31	4	3000	1. yes	90	10500	1	36	2. married	2. oly read
43		3300	1. yes	100	14600	6	27	2. married	4. grade 9-
17	3		1. yes	6	9400	7	30	2. married	2. oly read
39	1		1. yes	73	9000	3	22	2. married	2. oly read
50		3900	1. yes	98	15200	6	26	2. married	5. college (
7	0	3500	1. yes	70	7500	5	25	2. married	5. college (
9	1	3500	1. yes	67	7500	5	30	2. married	5. college (
36	0	3600	1. yes	104	15000	6	28	3. divorcec	5. college (
34	1	3500	1. yes	97	12500	6	28	2. married	5. college (
11	0	2400	1. yes	70	8900	6	26	2. married	3. grade 1-
25	1	4500	1. yes	85	12000	6	32	2. married	2. oly read
30	6	3100	1. yes	84	10900	6	35	2. married	2. oly read
20	2	3000	1. yes	84	10500	9	23	2. married	4. grade 9-
30		3000	1. yes	89	12100	6	23	2. married	4. grade 9-
25	3	4500	1. yes	89	12500	6	34	2. married	2. oly read
53	0	3700	1. yes	102	14400	6	30	2. married	4. grade 9-
42	3	31000	1. yes	101	14100	6	45	2. married	2. oly read
54	0	3400	1. yes	102	15100	7	28	2. married	5. college (
59	3		1. yes	110	17000	6	35		3. grade 1-
11	0	3500	1. yes	72	9400	6	19	2. married	3. grade 1-
10	1	3000	1. yes	3	8000	6	28	2. married	4. grade 9-
30	6	3100	1. yes	84	10900	6	35	2. married	2. oly read
8	1	3750	1. yes	72	9000	6	32	2. married	5. college (
8	1	2100	1. yes	71	9900	6	32	2. married	3. grade 1-

39	2		1. yes	88	11000	8	33	4. widowec	3. grade 1-
31	2		1. yes	91	12000	6	32	2. married	4. grade 9-
56	2	3500	1. yes	99	14900	6	29	2. married	5. college
31	0	3000	1. yes	88	11300	6	30	2. married	5. college
25	3	4500	1. yes	89	12500	6	34	2. married	2. oly read
34	0		1. yes	89	11300	6	28	2. married	4. grade 9-
10	0	3500	1. yes	73	9500	6	26	3. divorcec	5. college
11	1		1. yes	69	7800	7	35	2. married	4. grade 9-
36	0	2900	1. yes	100	13100	6	25	2. married	3. grade 1-
59	1		1. yes	115	19500	8	35	2. married	2. oly read
59	0	1800	1. yes	110	5	30	30	2. married	3. grade 1-
43		3300	1. yes	100	14600	6	27	2. married	4. grade 9-
17	3		1. yes	6	9400	7	30	2. married	2. oly read
13	1		1. yes	71	9000	8	30	2. married	2. oly read
11	0	3500	1. yes	73	8800	6	25	2. married	5. college
25	1	3000	1. yes	89	15200	6	35	2. married	5. college
53	1	3500	1. yes	110	16.5	6	35	2. married	3. grade 1-
9	1	3500	1. yes	67	7500	5	30	2. married	5. college
36	0	3000	1. yes	105	14000	6	24	2. married	4. grade 9-
36	0	3500	1. yes	100	12000	6	26	2. married	5. college
10	0		1. yes	68	9000	6	22	3. divorcec	2. oly read
34	1	3500	1. yes	97	12500	6	28	2. married	5. college
25	1	4500	1. yes	85	12000	6	32	2. married	2. oly read
30	6	3100	1. yes	84	10900	6	35	2. married	2. oly read
11	0	3300	1. yes	1	8900	6	21	2. married	4. grade 9-
48	2	3000	1. yes	99	14400	6	35	2. married	2. oly read
25	3	4500	1. yes	89	12500	6	34	2. married	2. oly read
17	1	3000	1. yes	74	14000	6	28	2. married	3. grade 1-
28	0	3400	1. yes	98	1200	5	29	2. married	5. college
11	0	3500	1. yes	72	9400	6	19	2. married	3. grade 1-
43		3300	1. yes	100	14600	6	27	2. married	4. grade 9-
10	1	3000	1. yes	3	8000	6	28	2. married	4. grade 9-
30	6	3100	1. yes	84	10900	6	35	2. married	2. oly read
56	2	3500	1. yes	99	14900	6	29	2. married	5. college
14	0	3000	1. yes	6	9100	6	28	2. married	5. college
42	3	31000	1. yes	101	14100	6	45	2. married	2. oly read
8	1	3750	1. yes	72	9000	6	32	2. married	5. college
56	2	3500	1. yes	99	14900	6	29	2. married	5. college
25	3	4500	1. yes	89	12500	6	34	2. married	2. oly read
42	2	3000	1. yes	98	12000	6	26	2. married	3. grade 1-
28	0	3400	1. yes	98	1200	5	29	2. married	5. college
14	0	3000	1. yes	80	6	24	24	2. married	5. college
32	1	4000	1. yes	95	14100	6	30	2. married	3. grade 1-
43		3300	1. yes	100	14600	6	27	2. married	4. grade 9-
17	3		1. yes	6	9400	7	30	2. married	2. oly read
7	0	3500	1. yes	70	7500	5	25	2. married	5. college
36	0	3600	1. yes	104	15000	6	28	3. divorcec	5. college
34	1	3500	1. yes	97	12500	6	28	2. married	5. college
11	0	2400	1. yes	70	8900	6	26	2. married	3. grade 1-
30		3000	1. yes	89	12100	6	23	2. married	4. grade 9-
42	3	31000	1. yes	101	14100	6	45	2. married	2. oly read
54	0	3400	1. yes	102	15100	7	28	2. married	5. college

56	2	3500	1. yes	99	14900	6	29	2. married	5. college ‹
10	0	3500	1. yes	73	9500	6	26	3. divorcec	5. college ‹
42	2	3000	1. yes	98	12000	6	26	2. married	3. grade 1-
11	0		1. yes	72	8500	4	24	2. married	4. grade 9-
59	3		0. no	102	14400	7	35	2. married	2. oly read
11	0	3500	1. yes	72	9400	6	19	2. married	3. grade 1-
32	1	4000	1. yes	95	14100	6	30	2. married	3. grade 1-
50		3900	1. yes	98	15200	6	26	2. married	5. college ‹
25	1	3000	1. yes	89	15200	6	35	2. married	5. college ‹
7	0	3500	1. yes	70	7500	5	25	2. married	5. college ‹
36	0	3600	1. yes	104	15000	6	28	3. divorcec	5. college ‹
10	1	3000	1. yes	3	8000	6	28	2. married	4. grade 9-
56	2	3500	1. yes	99	14900	6	29	2. married	5. college ‹
53	0	3700	1. yes	102	14400	6	30	2. married	4. grade 9-
42	3	31000	1. yes	101	14100	6	45	2. married	2. oly read
54	0	3400	1. yes	102	15100	7	28	2. married	5. college ‹
10	0	3500	1. yes	73	9500	6	26	3. divorcec	5. college ‹
17	1	3000	1. yes	74	14000	6	28	2. married	3. grade 1-
43		3300	1. yes	100	14600	6	27	2. married	4. grade 9-
25	1	3000	1. yes	89	15200	6	35	2. married	5. college ‹
59		4000	1. yes	100	15300	24	37	2. married	3. grade 1-
31	2		1. yes	91	12000	6	32	2. married	4. grade 9-
11	0		1. yes	72	8500	4	24	2. married	4. grade 9-
17	3		1. yes	6	9400	7	30	2. married	2. oly read
42	3	31000	1. yes	101	14100	6	45	2. married	2. oly read
10	1	3000	1. yes	3	8000	6	28	2. married	4. grade 9-
39	0	3000	1. yes	80	11000	6	20	2. married	3. grade 1-
42	2	3000	1. yes	98	12000	6	26	2. married	3. grade 1-
28	0	3400	1. yes	98	1200	5	29	2. married	5. college ‹
14	0	3000	1. yes	80	6	24	24	2. married	5. college ‹
48	3		1. yes	98	14600	12	30	2. married	3. grade 1-
45	5	3000	1. yes	110	16600	3	35	2. married	2. oly read
43		3300	1. yes	100	14600	6	27	2. married	4. grade 9-
17	3		1. yes	6	9400	7	30	2. married	2. oly read
50		3900	1. yes	98	15200	6	26	2. married	5. college ‹
30	6	3100	1. yes	84	10900	6	35	2. married	2. oly read
8	1	3750	1. yes	72	9000	6	32	2. married	5. college ‹
8	1	2100	1. yes	71	9900	6	32	2. married	3. grade 1-
25	3	4500	1. yes	89	12500	6	34	2. married	2. oly read
34	0		1. yes	89	11300	6	28	2. married	4. grade 9-
10	0	3500	1. yes	73	9500	6	26	3. divorcec	5. college ‹
11	1		1. yes	69	7800	7	35	2. married	4. grade 9-
59	1		1. yes	115	19500	8	35	2. married	2. oly read
43		3300	1. yes	100	14600	6	27	2. married	4. grade 9-
36	0	3000	1. yes	105	14000	6	24	2. married	4. grade 9-
36	0	3500	1. yes	100	12000	6	26	2. married	5. college ‹
10	0		1. yes	68	9000	6	22	3. divorcec	2. oly read
25	1	4500	1. yes	85	12000	6	32	2. married	2. oly read
56	2	3500	1. yes	99	14900	6	29	2. married	5. college ‹
32	1	4000	1. yes	95	14100	6	30	2. married	3. grade 1-
53	0	3700	1. yes	102	14400	6	30	2. married	4. grade 9-
42	2	3000	1. yes	98	12000	6	26	2. married	3. grade 1-

39	2		1. yes	88	11000	8	33	4. widowec	3. grade 1-
36	0	2900	1. yes	100	13100	6	25	2. married	3. grade 1-
59	0	1800	1. yes	110	5	30	30	2. married	3. grade 1-
7	2	2800	1. yes	65	8000	6	32	2. married	2. oly read
30	6	3100	1. yes	84	10900	6	35	2. married	2. oly read
48	2	3000	1. yes	99	14400	6	35	2. married	2. oly read
25	3	4500	1. yes	89	12500	6	34	2. married	2. oly read
28	0	3400	1. yes	98	1200	5	29	2. married	5. college (
59		4000	1. yes	100	15300	24	37	2. married	3. grade 1-
30	6	3100	1. yes	84	10900	6	35	2. married	2. oly read
14	0	3000	1. yes	6	9100	6	28	2. married	5. college (
53	1	3500	1. yes	110	16.5	6	35	2. married	3. grade 1-
42	3	31000	1. yes	101	14100	6	45	2. married	2. oly read
10	1	3000	1. yes	3	8000	6	28	2. married	4. grade 9-
8	1	3750	1. yes	72	9000	6	32	2. married	5. college (
8	1	2100	1. yes	71	9900	6	32	2. married	3. grade 1-
42	2	3000	1. yes	98	12000	6	26	2. married	3. grade 1-
11	1		1. yes	69	7800	7	35	2. married	4. grade 9-
59	3		0. no	102	14400	7	35	2. married	2. oly read
43		3300	1. yes	100	14600	6	27	2. married	4. grade 9-
25	1	4500	1. yes	85	12000	6	32	2. married	2. oly read
59	3		1. yes	110	17000	6	35		3. grade 1-
10	0	3500	1. yes	73	9500	6	26	3. divorcec	5. college (
36	0	2900	1. yes	100	13100	6	25	2. married	3. grade 1-
59	0	1800	1. yes	110	5	30	30	2. married	3. grade 1-
43		3300	1. yes	100	14600	6	27	2. married	4. grade 9-
9	1	3500	1. yes	67	7500	5	30	2. married	5. college (
25	1	4500	1. yes	85	12000	6	32	2. married	2. oly read
30	6	3100	1. yes	84	10900	6	35	2. married	2. oly read
48	2	3000	1. yes	99	14400	6	35	2. married	2. oly read
17	1	3000	1. yes	74	14000	6	28	2. married	3. grade 1-
11	0	3500	1. yes	72	9400	6	19	2. married	3. grade 1-
43		3300	1. yes	100	14600	6	27	2. married	4. grade 9-
25	3	4500	1. yes	89	12500	6	34	2. married	2. oly read
14	0	3000	1. yes	80	6	24	24	2. married	5. college (
17	3		1. yes	6	9400	7	30	2. married	2. oly read
34	1	3500	1. yes	97	12500	6	28	2. married	5. college (
25	1	3000	1. yes	89	15200	6	35	2. married	5. college (
10	1	3000	1. yes	3	8000	6	28	2. married	4. grade 9-
56	2	3500	1. yes	99	14900	6	29	2. married	5. college (
10	0	3500	1. yes	73	9500	6	26	3. divorcec	5. college (
17	1	3000	1. yes	74	14000	6	28	2. married	3. grade 1-
43		3300	1. yes	100	14600	6	27	2. married	4. grade 9-
25	1	3000	1. yes	89	15200	6	35	2. married	5. college (
59		4000	1. yes	100	15300	24	37	2. married	3. grade 1-
14	0	3000	1. yes	80	6	24	24	2. married	5. college (
10	0	3500	1. yes	73	9500	6	26	3. divorcec	5. college (
11	1		1. yes	69	7800	7	35	2. married	4. grade 9-
11	0	3300	1. yes	1	8900	6	21	2. married	4. grade 9-
48	2	3000	1. yes	99	14400	6	35	2. married	2. oly read
56	2	3500	1. yes	99	14900	6	29	2. married	5. college (
54	0	3400	1. yes	102	15100	7	28	2. married	5. college (

10	0	3500	1. yes	73	9500	6	26	3. divorcec	5. college ‹
8	1	2100	1. yes	71	9900	6	32	2. married	3. grade 1-
31	2		1. yes	91	12000	6	32	2. married	4. grade 9-
11	1		1. yes	69	7800	7	35	2. married	4. grade 9-
59	3		1. yes	110	17000	6	35		3. grade 1-
36	0	2900	1. yes	100	13100	6	25	2. married	3. grade 1-
17	1	3000	1. yes	74	14000	6	28	2. married	3. grade 1-
59	3		0. no	102	14400	7	35	2. married	2. oly read
18	1	2500	1. yes	81	10000	5	30	2. married	5. college ‹
14	0	3000	1. yes	6	9100	6	28	2. married	5. college ‹
45	5	3000	1. yes	110	16600	3	35	2. married	2. oly read
17	3		1. yes	6	9400	7	30	2. married	2. oly read
39	1		1. yes	73	9000	3	22	2. married	2. oly read
11	0	3500	1. yes	73	8800	6	25	2. married	5. college ‹
36	0	3000	1. yes	105	14000	6	24	2. married	4. grade 9-
36	0	3600	1. yes	104	15000	6	28	3. divorcec	5. college ‹
10	0		1. yes	68	9000	6	22	3. divorcec	2. oly read
34	1	3500	1. yes	97	12500	6	28	2. married	5. college ‹
59		4000	1. yes	100	15300	24	37	2. married	3. grade 1-
29	0	3200	1. yes	95	12000	6	31	2. married	5. college ‹
20	2	3000	1. yes	84	10500	9	23	2. married	4. grade 9-
48	2	3000	1. yes	99	14400	6	35	2. married	2. oly read
25	3	4500	1. yes	89	12500	6	34	2. married	2. oly read
53	0	3700	1. yes	102	14400	6	30	2. married	4. grade 9-
11	1		1. yes	69	7800	7	35	2. married	4. grade 9-
59	3		1. yes	110	17000	6	35		3. grade 1-
17	1	3000	1. yes	74	14000	6	28	2. married	3. grade 1-
25	1	3000	1. yes	89	15200	6	35	2. married	5. college ‹
59		4000	1. yes	100	15300	24	37	2. married	3. grade 1-
30	6	3100	1. yes	84	10900	6	35	2. married	2. oly read
8	1	3750	1. yes	72	9000	6	32	2. married	5. college ‹
31	2		1. yes	91	12000	6	32	2. married	4. grade 9-
29	0		1. yes	84	10500	6	25	2. married	5. college ‹
36	0	2900	1. yes	100	13100	6	25	2. married	3. grade 1-
17	3		1. yes	6	9400	7	30	2. married	2. oly read
9	1	3500	1. yes	67	7500	5	30	2. married	5. college ‹
42	3	31000	1. yes	101	14100	6	45	2. married	2. oly read
10	1	3000	1. yes	3	8000	6	28	2. married	4. grade 9-
39	0	3000	1. yes	80	11000	6	20	2. married	3. grade 1-
8	1	3750	1. yes	72	9000	6	32	2. married	5. college ‹
48	2	3000	1. yes	99	14400	6	35	2. married	2. oly read
56	2	3500	1. yes	99	14900	6	29	2. married	5. college ‹
31	0	3000	1. yes	88	11300	6	30	2. married	5. college ‹
53	0	3700	1. yes	102	14400	6	30	2. married	4. grade 9-
54	0	3400	1. yes	102	15100	7	28	2. married	5. college ‹
8	1	2100	1. yes	71	9900	6	32	2. married	3. grade 1-
11	1		1. yes	69	7800	7	35	2. married	4. grade 9-
45	5	3000	1. yes	110	16600	3	35	2. married	2. oly read
39	1		1. yes	73	9000	3	22	2. married	2. oly read
50		3900	1. yes	98	15200	6	26	2. married	5. college ‹
9	1	3500	1. yes	67	7500	5	30	2. married	5. college ‹
36	0	3600	1. yes	104	15000	6	28	3. divorcec	5. college ‹

25	1	4500	1. yes	85	12000	6	32	2. married	2. oly read
30	6	3100	1. yes	84	10900	6	35	2. married	2. oly read
53	0	3700	1. yes	102	14400	6	30	2. married	4. grade 9-
42	3	31000	1. yes	101	14100	6	45	2. married	2. oly read
39	2		1. yes	88	11000	8	33	4. widowec	3. grade 1-
31	2		1. yes	91	12000	6	32	2. married	4. grade 9-
31	0	3000	1. yes	88	11300	6	30	2. married	5. college
25	3	4500	1. yes	89	12500	6	34	2. married	2. oly read
10	0	3500	1. yes	73	9500	6	26	3. divorcec	5. college
59	0	1800	1. yes	110	5	30	30	2. married	3. grade 1-
17	3		1. yes	6	9400	7	30	2. married	2. oly read
36	0	3000	1. yes	105	14000	6	24	2. married	4. grade 9-
25	1	4500	1. yes	85	12000	6	32	2. married	2. oly read
30	6	3100	1. yes	84	10900	6	35	2. married	2. oly read
28	0	3400	1. yes	98	1200	5	29	2. married	5. college
43		3300	1. yes	100	14600	6	27	2. married	4. grade 9-
56	2	3500	1. yes	99	14900	6	29	2. married	5. college
42	3	31000	1. yes	101	14100	6	45	2. married	2. oly read
8	1	3750	1. yes	72	9000	6	32	2. married	5. college
25	3	4500	1. yes	89	12500	6	34	2. married	2. oly read
43		3300	1. yes	100	14600	6	27	2. married	4. grade 9-
30		3000	1. yes	89	12100	6	23	2. married	4. grade 9-
56	2	3500	1. yes	99	14900	6	29	2. married	5. college
32	1	4000	1. yes	95	14100	6	30	2. married	3. grade 1-
25	1	3000	1. yes	89	15200	6	35	2. married	5. college
42	3	31000	1. yes	101	14100	6	45	2. married	2. oly read
14	0	3000	1. yes	80	6	24	24	2. married	5. college
8	1	3750	1. yes	72	9000	6	32	2. married	5. college
11	1		1. yes	69	7800	7	35	2. married	4. grade 9-
36	0	3000	1. yes	105	14000	6	24	2. married	4. grade 9-
25	1	4500	1. yes	85	12000	6	32	2. married	2. oly read
32	1	4000	1. yes	95	14100	6	30	2. married	3. grade 1-
53	0	3700	1. yes	102	14400	6	30	2. married	4. grade 9-
42	2	3000	1. yes	98	12000	6	26	2. married	3. grade 1-
39	2		1. yes	88	11000	8	33	4. widowec	3. grade 1-
28	0	3400	1. yes	98	1200	5	29	2. married	5. college
8	1	3750	1. yes	72	9000	6	32	2. married	5. college
8	1	2100	1. yes	71	9900	6	32	2. married	3. grade 1-
43		3300	1. yes	100	14600	6	27	2. married	4. grade 9-
59	0	1800	1. yes	110	5	30	30	2. married	3. grade 1-
43		3300	1. yes	100	14600	6	27	2. married	4. grade 9-
43		3300	1. yes	100	14600	6	27	2. married	4. grade 9-
25	3	4500	1. yes	89	12500	6	34	2. married	2. oly read
34	1	3500	1. yes	97	12500	6	28	2. married	5. college
10	1	3000	1. yes	3	8000	6	28	2. married	4. grade 9-
43		3300	1. yes	100	14600	6	27	2. married	4. grade 9-
59		4000	1. yes	100	15300	24	37	2. married	3. grade 1-
10	0	3500	1. yes	73	9500	6	26	3. divorcec	5. college
11	0	3300	1. yes	1	8900	6	21	2. married	4. grade 9-
30	6	3100	1. yes	84	10900	6	35	2. married	2. oly read
28	0	3400	1. yes	98	1200	5	29	2. married	5. college
11	0	3500	1. yes	72	9400	6	19	2. married	3. grade 1-

48	2	3000	1. yes	99	14400	6	35	2. married	2. oly read

4. grade 9-	5. house w	1. amhara	1. rural	0. no	1. iodized	1. yes	42.8		0. no
3. grade 1-	5. house w	1. amhara	1. rural	1. yes	0. non iodi	1. yes	38.5		0. no
5. college	1. goverme	1. amhara	2. urban	0. no	1. iodized	1. yes	38.8	0.6	0. no
4. grade 9-	5. house w	1. amhara	2. urban	0. no	1. iodized	1. yes	38.5	8	0. no
5. college	2. merchat	1. amhara	2. urban	0. no	1. iodized	1. yes	38.8	2	0. no
4. grade 9-	5. house w	1. amhara	2. urban	0. no	1. iodized	1. yes	38.5	5	0. no
5. college	1. goverme	1. amhara	2. urban	0. no	1. iodized	0. no	38.5		0. no
3. grade 1-	2. merchat	1. amhara	2. urban	0. no	0. non iodi	0. no	42.8	11	0. no
3. grade 1-	5. house w	1. amhara	2. urban	0. no	1. iodized	1. yes	38.5	3	0. no
5. college	5. house w	1. amhara	2. urban	0. no	1. iodized	0. no	38.8		0. no
3. grade 1-	5. house w	1. amhara	1. rural	1. yes	1. iodized	1. yes	38.5	8	0. no
5. college	1. goverme	1. amhara	1. rural	0. no	1. iodized	1. yes	38.5		0. no
4. grade 9-	5. house w	1. amhara	2. urban	0. no	1. iodized	1. yes	38.5		0. no
3. grade 1-	5. house w	1. amhara	2. urban	0. no	1. iodized	1. yes	38.5		0. no
4. grade 9-	5. house w	1. amhara	2. urban	0. no	1. iodized	1. yes	35	13	1. yes
3. grade 1-	5. house w	1. amhara	1. rural	0. no	0. non iodi	1. yes	40	10	0. no
2. oly read	5. house w	1. amhara	2. urban	0. no	0. non iodi	1. yes	38.5	10	0. no
4. grade 9-	5. house w	1. amhara	1. rural	0. no	0. non iodi	1. yes	38.5	2	0. no
5. college	1. goverme	1. amhara	2. urban	0. no	0. non iodi	1. yes	38.5		0. no
4. grade 9-	2. merchat	1. amhara	2. urban	0. no	1. iodized	0. no	38.6	3.5	0. no
3. grade 1-	1. goverme	1. amhara	1. rural	0. no	1. iodized	1. yes	38.5	4	0. no
3. grade 1-	5. house w	1. amhara	1. rural	0. no		0. no			0. no
4. grade 9-	1. goverme	1. amhara	2. urban	0. no	1. iodized	1. yes	38.1	10	0. no
4. grade 9-	5. house w	1. amhara	2. urban	0. no	1. iodized	1. yes	37	4	0. no
5. college	1. goverme	1. amhara	2. urban	0. no	1. iodized	1. yes	38		0. no
3. grade 1-	5. house w	1. amhara	2. urban	0. no	0. non iodi	0. no			1. yes
5. college	5. house w	1. amhara	2. urban	1. yes	1. iodized	1. yes	38.5	1	0. no
2. oly read	6. others	1. amhara	1. rural	0. no	0. non iodi	0. no	38.5	2	0. no
5. college	1. goverme	1. amhara	2. urban	0. no	1. iodized	1. yes	37	1.3	0. no
3. grade 1-	5. house w	1. amhara	2. urban	0. no	0. non iodi	0. no	38.5	3	1. yes
5. college	1. goverme	1. amhara	2. urban	0. no	1. iodized	1. yes	40	0	0. no
4. grade 9-	5. house w	1. amhara	2. urban	0. no	1. iodized	1. yes	39		0. no
5. college	5. house w	1. amhara	2. urban	0. no	1. iodized	1. yes	42	0	0. no
4. grade 9-	5. house w	2. oromo	2. urban	0. no	1. iodized	1. yes	41		0. no
2. oly read	5. house w	1. amhara	1. rural	0. no	1. iodized	1. yes	38.5	4	0. no
3. grade 1-	2. merchat	1. amhara	2. urban	0. no	1. iodized	1. yes	38.5		0. no
2. oly read	5. house w	1. amhara	1. rural	0. no	0. non iodi	1. yes	37.3	2	0. no
3. grade 1-	5. house w	1. amhara	1. rural	0. no	0. non iodi	1. yes	40	10	0. no
3. grade 1-	2. merchat	1. amhara	2. urban	0. no		0. no	38.5	2	0. no
2. oly read	5. house w	1. amhara	2. urban	0. no	1. iodized	1. yes	38.5	5	1. yes
2. oly read	5. house w	1. amhara	1. rural	0. no	1. iodized	1. yes	38.5	6	0. no
2. oly read	5. house w	1. amhara	1. rural	0. no	1. iodized	0. no	38.5	9	0. no
4. grade 9-	5. house w	1. amhara	2. urban	0. no	1. iodized	0. no	38.5		0. no
2. oly read	5. house w	1. amhara	1. rural	0. no	0. non iodi	0. no	38.5	2	0. no
2. oly read	5. house w	1. amhara	1. rural	0. no	0. non iodi	1. yes	38.5	3	0. no
2. oly read	2. merchat	1. amhara	2. urban	0. no	1. iodized	1. yes	40.5	3	0. no
5. college	5. house w	1. amhara	2. urban	0. no	0. non iodi	1. yes	38.5		0. no
5. college	5. house w	1. amhara	2. urban	0. no	1. iodized	1. yes	40		0. no
5. college	1. goverme	1. amhara	2. urban	0. no	1. iodized	1. yes	39.5		0. no
5. college	1. goverme	1. amhara	2. urban	0. no	1. iodized	1. yes	38.5	2.75	0. no
5. college	5. house w	1. amhara	2. urban	1. yes	1. iodized	0. no	38.5	11	0. no
5. college	1. goverme	1. amhara	2. urban	0. no	1. iodized	1. yes	40		0. no

5. college	1. goverme	1. amhara	2. urban	0. no	1. iodized	1. yes	39	4	0. no
2. oly read	5. house w	1. amhara	2. urban	0. no	1. iodized	1. yes	36	7	0. no
3. grade 1-	2. merchat	2. oromo	2. urban	0. no	1. iodized	1. yes	39	2.5	0. no
5. college	1. goverme	1. amhara	2. urban	0. no		0. no			0. no
5. college	5. house w	1. amhara	2. urban	0. no	1. iodized	1. yes	38		0. no
3. grade 1-	5. house w	1. amhara	1. rural	0. no	0. non iodi	0. no	38.5		0. no
5. college	1. goverme	1. amhara	2. urban	0. no	1. iodized	0. no	40	2.6	0. no
3. grade 1-	3. private e	4. others	1. rural	1. yes	1. iodized	0. no	41	40	0. no
4. grade 9-	5. house w	1. amhara	1. rural	0. no	1. iodized	0. no	38.5		0. no
4. grade 9-	5. house w	1. amhara	2. urban	0. no	1. iodized	0. no	38.5		0. no
5. college	1. goverme	1. amhara	2. urban	0. no	1. iodized	1. yes	38		0. no
2. oly read	5. house w	1. amhara	2. urban	0. no	0. non iodi	1. yes	44	3	1. yes
5. college	5. house w	1. amhara	2. urban	1. yes	1. iodized	1. yes	38.5	3	1. yes
2. oly read	5. house w	1. amhara	1. rural	0. no	1. iodized	1. yes	39	2.5	0. no
5. college	5. house w	1. amhara	2. urban	0. no	1. iodized	1. yes	38.5	3	0. no
3. grade 1-	5. house w	1. amhara	2. urban	0. no	1. iodized	1. yes	38.5		0. no
4. grade 9-	5. house w	1. amhara	1. rural	0. no	1. iodized	1. yes	42.8		0. no
4. grade 9-	5. house w	1. amhara	2. urban	0. no	1. iodized	1. yes	38.5	8	0. no
5. college	2. merchat	1. amhara	2. urban	0. no	1. iodized	1. yes	38.8	2	0. no
3. grade 1-	5. house w	1. amhara	2. urban	0. no	1. iodized	1. yes	38.5	3	0. no
5. college	5. house w	1. amhara	2. urban	0. no	1. iodized	0. no	38.8		0. no
3. grade 1-	5. house w	1. amhara	1. rural	1. yes	1. iodized	1. yes	38.5	8	0. no
5. college	1. goverme	1. amhara	1. rural	0. no	1. iodized	1. yes	38.5		0. no
3. grade 1-	5. house w	1. amhara	2. urban	0. no	1. iodized	1. yes	38.5		0. no
4. grade 9-	2. merchat	1. amhara	2. urban	0. no	1. iodized	0. no	38.6	3.5	0. no
3. grade 1-	1. goverme	1. amhara	1. rural	0. no	1. iodized	1. yes	38.5	4	0. no
4. grade 9-	5. house w	1. amhara	2. urban	0. no	1. iodized	1. yes	37	4	0. no
5. college	1. goverme	1. amhara	2. urban	0. no	1. iodized	1. yes	37	1.3	0. no
5. college	1. goverme	1. amhara	2. urban	0. no	1. iodized	1. yes	40	0	0. no
4. grade 9-	5. house w	1. amhara	2. urban	0. no	1. iodized	1. yes	39		0. no
4. grade 9-	5. house w	2. oromo	2. urban	0. no	1. iodized	1. yes	41		0. no
4. grade 9-	5. house w	1. amhara	2. urban	0. no	1. iodized	0. no	38.5		0. no
5. college	1. goverme	1. amhara	2. urban	0. no	1. iodized	1. yes	38.5	2.75	0. no
3. grade 1-	3. private e	4. others	1. rural	1. yes	1. iodized	0. no	41	40	0. no
5. college	5. house w	1. amhara	2. urban	1. yes	1. iodized	1. yes	38.5	3	1. yes
2. oly read	5. house w	1. amhara	1. rural	0. no	1. iodized	1. yes	39	2.5	0. no
5. college	1. goverme	1. amhara	2. urban	0. no	1. iodized	1. yes	38.8	0.6	0. no
5. college	2. merchat	1. amhara	2. urban	0. no	1. iodized	1. yes	38.8	2	0. no
4. grade 9-	5. house w	1. amhara	2. urban	0. no	1. iodized	1. yes	35	13	1. yes
2. oly read	5. house w	1. amhara	2. urban	0. no	0. non iodi	1. yes	38.5	10	0. no
4. grade 9-	5. house w	1. amhara	1. rural	0. no	0. non iodi	1. yes	38.5	2	0. no
5. college	1. goverme	1. amhara	2. urban	0. no	0. non iodi	1. yes	38.5		0. no
3. grade 1-	5. house w	1. amhara	1. rural	0. no		0. no			0. no
4. grade 9-	1. goverme	1. amhara	2. urban	0. no	1. iodized	1. yes	38.1	10	0. no
4. grade 9-	5. house w	1. amhara	2. urban	0. no	1. iodized	1. yes	37	4	0. no
5. college	1. goverme	1. amhara	2. urban	0. no	1. iodized	1. yes	38		0. no
4. grade 9-	5. house w	1. amhara	2. urban	0. no	1. iodized	1. yes	39		0. no
4. grade 9-	5. house w	1. amhara	2. urban	0. no	1. iodized	0. no	38.5		0. no
2. oly read	5. house w	1. amhara	1. rural	0. no	0. non iodi	0. no	38.5	2	0. no
5. college	5. house w	1. amhara	2. urban	1. yes	1. iodized	0. no	38.5	11	0. no
5. college	1. goverme	1. amhara	2. urban	0. no	1. iodized	1. yes	39	4	0. no
5. college	1. goverme	1. amhara	2. urban	0. no	1. iodized	1. yes	38		0. no

3. grade 1-	5. house w	1. amhara	1. rural	1. yes	1. iodized	1. yes	38.5	8	0. no
5. college (	1. goverme	1. amhara	1. rural	0. no	1. iodized	1. yes	38.5		0. no
5. college (	5. house w	1. amhara	2. urban	1. yes	1. iodized	1. yes	38.5	3	1. yes
2. oly read	5. house w	1. amhara	1. rural	0. no	1. iodized	1. yes	39	2.5	0. no
4. grade 9-	5. house w	1. amhara	1. rural	0. no	1. iodized	1. yes	42.8		0. no
3. grade 1-	5. house w	1. amhara	1. rural	1. yes	0. non iodi:	1. yes	38.5		0. no
5. college (	1. goverme	1. amhara	2. urban	0. no	1. iodized	1. yes	38.8	0.6	0. no
4. grade 9-	5. house w	1. amhara	2. urban	0. no	1. iodized	1. yes	38.5	8	0. no
5. college (	2. merchat	1. amhara	2. urban	0. no	1. iodized	1. yes	38.8	2	0. no
5. college (	1. goverme	1. amhara	2. urban	0. no	1. iodized	0. no	38.5		0. no
3. grade 1-	5. house w	1. amhara	2. urban	0. no	1. iodized	1. yes	38.5	3	0. no
5. college (	5. house w	1. amhara	2. urban	0. no	1. iodized	0. no	38.8		0. no
5. college (	1. goverme	1. amhara	1. rural	0. no	1. iodized	1. yes	38.5		0. no
3. grade 1-	5. house w	1. amhara	2. urban	0. no	1. iodized	1. yes	38.5		0. no
4. grade 9-	5. house w	1. amhara	2. urban	0. no	1. iodized	1. yes	35	13	1. yes
3. grade 1-	5. house w	1. amhara	1. rural	0. no	0. non iodi:	1. yes	40	10	0. no
2. oly read	5. house w	1. amhara	2. urban	0. no	0. non iodi:	1. yes	38.5	10	0. no
4. grade 9-	2. merchat	1. amhara	2. urban	0. no	1. iodized	0. no	38.6	3.5	0. no
4. grade 9-	5. house w	1. amhara	2. urban	0. no	1. iodized	1. yes	37	4	0. no
5. college (	1. goverme	1. amhara	2. urban	0. no	1. iodized	1. yes	38		0. no
2. oly read	6. others	1. amhara	1. rural	0. no	0. non iodi:	0. no	38.5	2	0. no
5. college (	1. goverme	1. amhara	2. urban	0. no	1. iodized	1. yes	40	0	0. no
4. grade 9-	5. house w	1. amhara	2. urban	0. no	1. iodized	1. yes	39		0. no
5. college (	5. house w	1. amhara	2. urban	0. no	1. iodized	1. yes	42	0	0. no
2. oly read	5. house w	1. amhara	1. rural	0. no	1. iodized	1. yes	38.5	4	0. no
2. oly read	5. house w	1. amhara	1. rural	0. no	0. non iodi:	1. yes	37.3	2	0. no
3. grade 1-	2. merchat	1. amhara	2. urban	0. no		0. no	38.5	2	0. no
2. oly read	5. house w	1. amhara	1. rural	0. no	1. iodized	1. yes	38.5	6	0. no
2. oly read	5. house w	1. amhara	1. rural	0. no	1. iodized	0. no	38.5	9	0. no
4. grade 9-	5. house w	1. amhara	2. urban	0. no	1. iodized	0. no	38.5		0. no
2. oly read	5. house w	1. amhara	1. rural	0. no	0. non iodi:	0. no	38.5	2	0. no
2. oly read	2. merchat	1. amhara	2. urban	0. no	1. iodized	1. yes	40.5	3	0. no
5. college (	5. house w	1. amhara	2. urban	0. no	1. iodized	1. yes	40		0. no
5. college (	1. goverme	1. amhara	2. urban	0. no	1. iodized	1. yes	40		0. no
5. college (	1. goverme	1. amhara	2. urban	0. no	1. iodized	1. yes	39	4	0. no
5. college (	1. goverme	1. amhara	2. urban	0. no		0. no			0. no
5. college (	1. goverme	1. amhara	2. urban	0. no	1. iodized	0. no	40	2.6	0. no
4. grade 9-	5. house w	1. amhara	2. urban	0. no	1. iodized	0. no	38.5		0. no
2. oly read	5. house w	1. amhara	2. urban	0. no	0. non iodi:	1. yes	44	3	1. yes
2. oly read	5. house w	1. amhara	1. rural	0. no	1. iodized	1. yes	39	2.5	0. no
5. college (	5. house w	1. amhara	2. urban	0. no	1. iodized	1. yes	38.5	3	0. no
3. grade 1-	5. house w	1. amhara	2. urban	0. no	1. iodized	1. yes	38.5		0. no
3. grade 1-	5. house w	1. amhara	2. urban	0. no	1. iodized	1. yes	38.5	3	0. no
5. college (	5. house w	1. amhara	2. urban	0. no	1. iodized	0. no	38.8		0. no
3. grade 1-	5. house w	1. amhara	1. rural	1. yes	1. iodized	1. yes	38.5	8	0. no
5. college (	1. goverme	1. amhara	1. rural	0. no	1. iodized	1. yes	38.5		0. no
3. grade 1-	1. goverme	1. amhara	1. rural	0. no	1. iodized	1. yes	38.5	4	0. no
4. grade 9-	5. house w	2. oromo	2. urban	0. no	1. iodized	1. yes	41		0. no
5. college (	5. house w	1. amhara	2. urban	1. yes	1. iodized	1. yes	38.5	3	1. yes
2. oly read	5. house w	1. amhara	1. rural	0. no	1. iodized	1. yes	39	2.5	0. no
5. college (	1. goverme	1. amhara	2. urban	0. no	1. iodized	1. yes	38.8	0.6	0. no
4. grade 9-	5. house w	1. amhara	2. urban	0. no	1. iodized	1. yes	35	13	1. yes

2. oly read	5. house w	1. amhara	2. urban	0. no	0. non iodi	1. yes	38.5	10	0. no
4. grade 9-	5. house w	1. amhara	1. rural	0. no	0. non iodi	1. yes	38.5	2	0. no
5. college	2. merchat	1. amhara	2. urban	0. no	1. iodized	1. yes	38.8	2	0. no
5. college	1. goverme	1. amhara	2. urban	0. no	1. iodized	0. no	38.5		0. no
3. grade 1-	5. house w	1. amhara	2. urban	0. no	1. iodized	1. yes	38.5	3	0. no
4. grade 9-	5. house w	1. amhara	2. urban	0. no	1. iodized	1. yes	38.5		0. no
3. grade 1-	5. house w	1. amhara	2. urban	0. no	1. iodized	1. yes	38.5		0. no
4. grade 9-	2. merchat	1. amhara	2. urban	0. no	1. iodized	0. no	38.6	3.5	0. no
3. grade 1-	5. house w	1. amhara	1. rural	0. no		0. no			0. no
3. grade 1-	5. house w	1. amhara	2. urban	0. no	0. non iodi	0. no			1. yes
3. grade 1-	2. merchat	1. amhara	2. urban	0. no	1. iodized	1. yes	38.5		0. no
4. grade 9-	5. house w	1. amhara	2. urban	0. no	1. iodized	0. no	38.5		0. no
2. oly read	5. house w	1. amhara	1. rural	0. no	0. non iodi	0. no	38.5	2	0. no
2. oly read	5. house w	1. amhara	1. rural	0. no	0. non iodi	1. yes	38.5	3	0. no
5. college	5. house w	1. amhara	2. urban	0. no	0. non iodi	1. yes	38.5		0. no
5. college	1. goverme	1. amhara	2. urban	0. no	1. iodized	1. yes	38.5	2.75	0. no
5. college	5. house w	1. amhara	2. urban	1. yes	1. iodized	0. no	38.5	11	0. no
5. college	1. goverme	1. amhara	2. urban	0. no	1. iodized	1. yes	39	4	0. no
3. grade 1-	2. merchat	2. oromo	2. urban	0. no	1. iodized	1. yes	39	2.5	0. no
5. college	5. house w	1. amhara	2. urban	0. no	1. iodized	1. yes	38		0. no
3. grade 1-	5. house w	1. amhara	1. rural	0. no	0. non iodi	0. no	38.5		0. no
5. college	1. goverme	1. amhara	2. urban	0. no	1. iodized	0. no	40	2.6	0. no
2. oly read	5. house w	1. amhara	2. urban	0. no	0. non iodi	1. yes	44	3	1. yes
2. oly read	5. house w	1. amhara	1. rural	0. no	1. iodized	1. yes	39	2.5	0. no
4. grade 9-	5. house w	1. amhara	1. rural	0. no	1. iodized	1. yes	42.8		0. no
4. grade 9-	5. house w	1. amhara	2. urban	0. no	1. iodized	1. yes	38.5	8	0. no
3. grade 1-	5. house w	1. amhara	2. urban	0. no	1. iodized	1. yes	38.5	3	0. no
4. grade 9-	5. house w	1. amhara	2. urban	0. no	1. iodized	1. yes	37	4	0. no
5. college	1. goverme	1. amhara	2. urban	0. no	1. iodized	1. yes	40	0	0. no
4. grade 9-	5. house w	2. oromo	2. urban	0. no	1. iodized	1. yes	41		0. no
4. grade 9-	5. house w	1. amhara	2. urban	0. no	1. iodized	0. no	38.5		0. no
5. college	5. house w	1. amhara	2. urban	1. yes	1. iodized	1. yes	38.5	3	1. yes
2. oly read	5. house w	1. amhara	1. rural	0. no	1. iodized	1. yes	39	2.5	0. no
5. college	2. merchat	1. amhara	2. urban	0. no	1. iodized	1. yes	38.8	2	0. no
4. grade 9-	5. house w	1. amhara	2. urban	0. no	1. iodized	1. yes	39		0. no
3. grade 1-	5. house w	1. amhara	1. rural	1. yes	1. iodized	1. yes	38.5	8	0. no
5. college	1. goverme	1. amhara	2. urban	0. no	1. iodized	1. yes	38.8	0.6	0. no
5. college	2. merchat	1. amhara	2. urban	0. no	1. iodized	1. yes	38.8	2	0. no
3. grade 1-	5. house w	1. amhara	2. urban	0. no	1. iodized	1. yes	38.5	3	0. no
3. grade 1-	5. house w	1. amhara	1. rural	0. no	0. non iodi	1. yes	40	10	0. no
5. college	1. goverme	1. amhara	2. urban	0. no	1. iodized	1. yes	40	0	0. no
5. college	5. house w	1. amhara	2. urban	0. no	1. iodized	1. yes	42	0	0. no
3. grade 1-	2. merchat	1. amhara	2. urban	0. no		0. no	38.5	2	0. no
4. grade 9-	5. house w	1. amhara	2. urban	0. no	1. iodized	0. no	38.5		0. no
2. oly read	5. house w	1. amhara	1. rural	0. no	0. non iodi	0. no	38.5	2	0. no
5. college	1. goverme	1. amhara	2. urban	0. no	1. iodized	1. yes	40		0. no
5. college	1. goverme	1. amhara	2. urban	0. no		0. no			0. no
5. college	1. goverme	1. amhara	2. urban	0. no	1. iodized	0. no	40	2.6	0. no
4. grade 9-	5. house w	1. amhara	2. urban	0. no	1. iodized	0. no	38.5		0. no
3. grade 1-	5. house w	1. amhara	2. urban	0. no	1. iodized	1. yes	38.5		0. no
3. grade 1-	5. house w	1. amhara	1. rural	1. yes	1. iodized	1. yes	38.5	8	0. no
5. college	1. goverme	1. amhara	1. rural	0. no	1. iodized	1. yes	38.5		0. no

5. college	2. merchat	1. amhara	2. urban	0. no	1. iodized	1. yes	38.8	2	0. no
3. grade 1-	5. house w	1. amhara	2. urban	0. no	1. iodized	1. yes	38.5		0. no
3. grade 1-	5. house w	1. amhara	1. rural	0. no	0. non iodi	1. yes	40	10	0. no
5. college	1. goverme	1. amhara	2. urban	0. no	1. iodized	1. yes	38		0. no
2. oly read	6. others	1. amhara	1. rural	0. no	0. non iodi	0. no	38.5	2	0. no
4. grade 9-	5. house w	2. oromo	2. urban	0. no	1. iodized	1. yes	41		0. no
3. grade 1-	2. merchat	1. amhara	2. urban	0. no		0. no	38.5	2	0. no
5. college	5. house w	1. amhara	2. urban	0. no	1. iodized	1. yes	40		0. no
5. college	1. goverme	1. amhara	2. urban	0. no	1. iodized	1. yes	38.5	2.75	0. no
5. college	1. goverme	1. amhara	2. urban	0. no	1. iodized	1. yes	40		0. no
5. college	1. goverme	1. amhara	2. urban	0. no		0. no			0. no
5. college	5. house w	1. amhara	2. urban	1. yes	1. iodized	1. yes	38.5	3	1. yes
5. college	2. merchat	1. amhara	2. urban	0. no	1. iodized	1. yes	38.8	2	0. no
5. college	5. house w	1. amhara	2. urban	0. no	1. iodized	0. no	38.8		0. no
3. grade 1-	5. house w	1. amhara	1. rural	1. yes	1. iodized	1. yes	38.5	8	0. no
5. college	1. goverme	1. amhara	1. rural	0. no	1. iodized	1. yes	38.5		0. no
3. grade 1-	5. house w	1. amhara	2. urban	0. no	1. iodized	1. yes	38.5		0. no
4. grade 9-	5. house w	1. amhara	2. urban	0. no	1. iodized	1. yes	37	4	0. no
4. grade 9-	5. house w	1. amhara	2. urban	0. no	1. iodized	0. no	38.5		0. no
5. college	1. goverme	1. amhara	2. urban	0. no	1. iodized	1. yes	38.5	2.75	0. no
3. grade 1-	3. private e	4. others	1. rural	1. yes	1. iodized	0. no	41	40	0. no
4. grade 9-	5. house w	1. amhara	1. rural	0. no	0. non iodi	1. yes	38.5	2	0. no
5. college	1. goverme	1. amhara	2. urban	0. no	1. iodized	1. yes	38		0. no
2. oly read	5. house w	1. amhara	1. rural	0. no	0. non iodi	0. no	38.5	2	0. no
3. grade 1-	5. house w	1. amhara	1. rural	1. yes	1. iodized	1. yes	38.5	8	0. no
5. college	5. house w	1. amhara	2. urban	1. yes	1. iodized	1. yes	38.5	3	1. yes
3. grade 1-	5. house w	1. amhara	1. rural	1. yes	0. non iodi	1. yes	38.5		0. no
3. grade 1-	5. house w	1. amhara	1. rural	0. no	0. non iodi	1. yes	40	10	0. no
5. college	1. goverme	1. amhara	2. urban	0. no	1. iodized	1. yes	40	0	0. no
5. college	5. house w	1. amhara	2. urban	0. no	1. iodized	1. yes	42	0	0. no
2. oly read	5. house w	1. amhara	1. rural	0. no	0. non iodi	1. yes	37.3	2	0. no
2. oly read	5. house w	1. amhara	1. rural	0. no	1. iodized	1. yes	38.5	6	0. no
4. grade 9-	5. house w	1. amhara	2. urban	0. no	1. iodized	0. no	38.5		0. no
2. oly read	5. house w	1. amhara	1. rural	0. no	0. non iodi	0. no	38.5	2	0. no
5. college	5. house w	1. amhara	2. urban	0. no	1. iodized	1. yes	40		0. no
2. oly read	5. house w	1. amhara	1. rural	0. no	1. iodized	1. yes	39	2.5	0. no
5. college	1. goverme	1. amhara	2. urban	0. no	1. iodized	1. yes	38.8	0.6	0. no
4. grade 9-	5. house w	1. amhara	2. urban	0. no	1. iodized	1. yes	35	13	1. yes
3. grade 1-	5. house w	1. amhara	2. urban	0. no	1. iodized	1. yes	38.5	3	0. no
4. grade 9-	5. house w	1. amhara	2. urban	0. no	1. iodized	1. yes	38.5		0. no
3. grade 1-	5. house w	1. amhara	2. urban	0. no	1. iodized	1. yes	38.5		0. no
4. grade 9-	2. merchat	1. amhara	2. urban	0. no	1. iodized	0. no	38.6	3.5	0. no
3. grade 1-	5. house w	1. amhara	2. urban	0. no	0. non iodi	0. no			1. yes
4. grade 9-	5. house w	1. amhara	2. urban	0. no	1. iodized	0. no	38.5		0. no
3. grade 1-	2. merchat	2. oromo	2. urban	0. no	1. iodized	1. yes	39	2.5	0. no
5. college	5. house w	1. amhara	2. urban	0. no	1. iodized	1. yes	38		0. no
3. grade 1-	5. house w	1. amhara	1. rural	0. no	0. non iodi	0. no	38.5		0. no
2. oly read	5. house w	1. amhara	2. urban	0. no	0. non iodi	1. yes	44	3	1. yes
5. college	2. merchat	1. amhara	2. urban	0. no	1. iodized	1. yes	38.8	2	0. no
3. grade 1-	2. merchat	1. amhara	2. urban	0. no		0. no	38.5	2	0. no
5. college	5. house w	1. amhara	2. urban	0. no	1. iodized	0. no	38.8		0. no
3. grade 1-	5. house w	1. amhara	1. rural	0. no	0. non iodi	1. yes	40	10	0. no

2. oly read	5. house w	1. amhara	2. urban	0. no	0. non iodi	1. yes	38.5	10	0. no
3. grade 1-	5. house w	1. amhara	1. rural	0. no		0. no			0. no
3. grade 1-	2. merchat	1. amhara	2. urban	0. no	1. iodized	1. yes	38.5		0. no
2. oly read	5. house w	1. amhara	2. urban	0. no	1. iodized	1. yes	36	7	0. no
2. oly read	5. house w	1. amhara	1. rural	0. no	1. iodized	1. yes	39	2.5	0. no
4. grade 9-	5. house w	1. amhara	2. urban	0. no	1. iodized	1. yes	38.5	8	0. no
3. grade 1-	5. house w	1. amhara	2. urban	0. no	1. iodized	1. yes	38.5	3	0. no
5. college	1. goverme	1. amhara	2. urban	0. no	1. iodized	1. yes	40	0	0. no
3. grade 1-	3. private e	4. others	1. rural	1. yes	1. iodized	0. no	41	40	0. no
2. oly read	5. house w	1. amhara	1. rural	0. no	1. iodized	1. yes	39	2.5	0. no
4. grade 9-	5. house w	1. amhara	2. urban	0. no	1. iodized	1. yes	39		0. no
5. college	5. house w	1. amhara	2. urban	1. yes	1. iodized	0. no	38.5	11	0. no
3. grade 1-	5. house w	1. amhara	1. rural	1. yes	1. iodized	1. yes	38.5	8	0. no
5. college	5. house w	1. amhara	2. urban	1. yes	1. iodized	1. yes	38.5	3	1. yes
5. college	1. goverme	1. amhara	2. urban	0. no	1. iodized	1. yes	38.8	0.6	0. no
4. grade 9-	5. house w	1. amhara	2. urban	0. no	1. iodized	1. yes	35	13	1. yes
3. grade 1-	5. house w	1. amhara	1. rural	0. no	0. non iodi	1. yes	40	10	0. no
4. grade 9-	2. merchat	1. amhara	2. urban	0. no	1. iodized	0. no	38.6	3.5	0. no
2. oly read	6. others	1. amhara	1. rural	0. no	0. non iodi	0. no	38.5	2	0. no
4. grade 9-	5. house w	1. amhara	2. urban	0. no	1. iodized	0. no	38.5		0. no
2. oly read	5. house w	1. amhara	2. urban	0. no	0. non iodi	1. yes	44	3	1. yes
3. grade 1-	1. goverme	1. amhara	1. rural	0. no	1. iodized	1. yes	38.5	4	0. no
3. grade 1-	5. house w	1. amhara	2. urban	0. no	1. iodized	1. yes	38.5		0. no
3. grade 1-	5. house w	1. amhara	1. rural	0. no		0. no			0. no
3. grade 1-	2. merchat	1. amhara	2. urban	0. no	1. iodized	1. yes	38.5		0. no
4. grade 9-	5. house w	1. amhara	2. urban	0. no	1. iodized	0. no	38.5		0. no
5. college	1. goverme	1. amhara	2. urban	0. no	1. iodized	1. yes	39	4	0. no
2. oly read	5. house w	1. amhara	2. urban	0. no	0. non iodi	1. yes	44	3	1. yes
2. oly read	5. house w	1. amhara	1. rural	0. no	1. iodized	1. yes	39	2.5	0. no
4. grade 9-	5. house w	1. amhara	2. urban	0. no	1. iodized	1. yes	38.5	8	0. no
4. grade 9-	5. house w	1. amhara	2. urban	0. no	1. iodized	1. yes	37	4	0. no
4. grade 9-	5. house w	2. oromo	2. urban	0. no	1. iodized	1. yes	41		0. no
4. grade 9-	5. house w	1. amhara	2. urban	0. no	1. iodized	0. no	38.5		0. no
3. grade 1-	5. house w	1. amhara	2. urban	0. no	1. iodized	1. yes	38.5	3	0. no
5. college	5. house w	1. amhara	2. urban	0. no	1. iodized	1. yes	42	0	0. no
2. oly read	5. house w	1. amhara	1. rural	0. no	0. non iodi	0. no	38.5	2	0. no
5. college	1. goverme	1. amhara	2. urban	0. no	1. iodized	0. no	40	2.6	0. no
5. college	1. goverme	1. amhara	2. urban	0. no	1. iodized	1. yes	38.5	2.75	0. no
5. college	5. house w	1. amhara	2. urban	1. yes	1. iodized	1. yes	38.5	3	1. yes
5. college	2. merchat	1. amhara	2. urban	0. no	1. iodized	1. yes	38.8	2	0. no
3. grade 1-	5. house w	1. amhara	2. urban	0. no	1. iodized	1. yes	38.5		0. no
4. grade 9-	5. house w	1. amhara	2. urban	0. no	1. iodized	1. yes	37	4	0. no
4. grade 9-	5. house w	1. amhara	2. urban	0. no	1. iodized	0. no	38.5		0. no
5. college	1. goverme	1. amhara	2. urban	0. no	1. iodized	1. yes	38.5	2.75	0. no
3. grade 1-	3. private e	4. others	1. rural	1. yes	1. iodized	0. no	41	40	0. no
5. college	5. house w	1. amhara	2. urban	0. no	1. iodized	1. yes	42	0	0. no
3. grade 1-	5. house w	1. amhara	2. urban	0. no	1. iodized	1. yes	38.5		0. no
4. grade 9-	2. merchat	1. amhara	2. urban	0. no	1. iodized	0. no	38.6	3.5	0. no
4. grade 9-	5. house w	1. amhara	1. rural	0. no	1. iodized	1. yes	42.8		0. no
4. grade 9-	5. house w	1. amhara	2. urban	0. no	1. iodized	1. yes	38.5	8	0. no
5. college	2. merchat	1. amhara	2. urban	0. no	1. iodized	1. yes	38.8	2	0. no
5. college	1. goverme	1. amhara	1. rural	0. no	1. iodized	1. yes	38.5		0. no

3. grade 1-	5. house w	1. amhara	2. urban	0. no	1. iodized	1. yes	38.5		0. no
4. grade 9-	5. house w	1. amhara	2. urban	0. no	1. iodized	1. yes	35	13	1. yes
4. grade 9-	5. house w	1. amhara	1. rural	0. no	0. non iodi	1. yes	38.5	2	0. no
4. grade 9-	2. merchat	1. amhara	2. urban	0. no	1. iodized	0. no	38.6	3.5	0. no
3. grade 1-	1. goverme	1. amhara	1. rural	0. no	1. iodized	1. yes	38.5	4	0. no
3. grade 1-	5. house w	1. amhara	1. rural	0. no		0. no			0. no
4. grade 9-	5. house w	1. amhara	2. urban	0. no	1. iodized	1. yes	37	4	0. no
2. oly read	6. others	1. amhara	1. rural	0. no	0. non iodi	0. no	38.5	2	0. no
5. college	1. goverme	1. amhara	2. urban	0. no	1. iodized	1. yes	37	1.3	0. no
4. grade 9-	5. house w	1. amhara	2. urban	0. no	1. iodized	1. yes	39		0. no
2. oly read	5. house w	1. amhara	1. rural	0. no	1. iodized	1. yes	38.5	6	0. no
2. oly read	5. house w	1. amhara	1. rural	0. no	0. non iodi	0. no	38.5	2	0. no
2. oly read	2. merchat	1. amhara	2. urban	0. no	1. iodized	1. yes	40.5	3	0. no
5. college	5. house w	1. amhara	2. urban	0. no	0. non iodi	1. yes	38.5		0. no
3. grade 1-	2. merchat	2. oromo	2. urban	0. no	1. iodized	1. yes	39	2.5	0. no
5. college	1. goverme	1. amhara	2. urban	0. no		0. no			0. no
3. grade 1-	5. house w	1. amhara	1. rural	0. no	0. non iodi	0. no	38.5		0. no
5. college	1. goverme	1. amhara	2. urban	0. no	1. iodized	0. no	40	2.6	0. no
3. grade 1-	3. private e	4. others	1. rural	1. yes	1. iodized	0. no	41	40	0. no
5. college	1. goverme	1. amhara	2. urban	0. no	1. iodized	1. yes	38		0. no
5. college	5. house w	1. amhara	2. urban	0. no	1. iodized	1. yes	38.5	3	0. no
4. grade 9-	5. house w	1. amhara	2. urban	0. no	1. iodized	1. yes	38.5	8	0. no
3. grade 1-	5. house w	1. amhara	2. urban	0. no	1. iodized	1. yes	38.5	3	0. no
5. college	5. house w	1. amhara	2. urban	0. no	1. iodized	0. no	38.8		0. no
4. grade 9-	2. merchat	1. amhara	2. urban	0. no	1. iodized	0. no	38.6	3.5	0. no
3. grade 1-	1. goverme	1. amhara	1. rural	0. no	1. iodized	1. yes	38.5	4	0. no
4. grade 9-	5. house w	1. amhara	2. urban	0. no	1. iodized	1. yes	37	4	0. no
5. college	1. goverme	1. amhara	2. urban	0. no	1. iodized	1. yes	38.5	2.75	0. no
3. grade 1-	3. private e	4. others	1. rural	1. yes	1. iodized	0. no	41	40	0. no
2. oly read	5. house w	1. amhara	1. rural	0. no	1. iodized	1. yes	39	2.5	0. no
5. college	1. goverme	1. amhara	2. urban	0. no	1. iodized	1. yes	38.8	0.6	0. no
4. grade 9-	5. house w	1. amhara	1. rural	0. no	0. non iodi	1. yes	38.5	2	0. no
5. college	1. goverme	1. amhara	2. urban	0. no	0. non iodi	1. yes	38.5		0. no
3. grade 1-	5. house w	1. amhara	1. rural	0. no		0. no			0. no
2. oly read	5. house w	1. amhara	1. rural	0. no	0. non iodi	0. no	38.5	2	0. no
5. college	1. goverme	1. amhara	2. urban	0. no	1. iodized	1. yes	39	4	0. no
3. grade 1-	5. house w	1. amhara	1. rural	1. yes	1. iodized	1. yes	38.5	8	0. no
5. college	5. house w	1. amhara	2. urban	1. yes	1. iodized	1. yes	38.5	3	1. yes
3. grade 1-	5. house w	1. amhara	1. rural	1. yes	0. non iodi	1. yes	38.5		0. no
5. college	1. goverme	1. amhara	2. urban	0. no	1. iodized	1. yes	38.8	0.6	0. no
4. grade 9-	5. house w	1. amhara	2. urban	0. no	1. iodized	1. yes	38.5	8	0. no
5. college	2. merchat	1. amhara	2. urban	0. no	1. iodized	1. yes	38.8	2	0. no
5. college	1. goverme	1. amhara	2. urban	0. no	1. iodized	0. no	38.5		0. no
5. college	5. house w	1. amhara	2. urban	0. no	1. iodized	0. no	38.8		0. no
5. college	1. goverme	1. amhara	1. rural	0. no	1. iodized	1. yes	38.5		0. no
4. grade 9-	5. house w	1. amhara	2. urban	0. no	1. iodized	1. yes	35	13	1. yes
4. grade 9-	2. merchat	1. amhara	2. urban	0. no	1. iodized	0. no	38.6	3.5	0. no
2. oly read	5. house w	1. amhara	1. rural	0. no	1. iodized	1. yes	38.5	6	0. no
2. oly read	2. merchat	1. amhara	2. urban	0. no	1. iodized	1. yes	40.5	3	0. no
5. college	5. house w	1. amhara	2. urban	0. no	1. iodized	1. yes	40		0. no
5. college	1. goverme	1. amhara	2. urban	0. no	1. iodized	1. yes	39	4	0. no
5. college	1. goverme	1. amhara	2. urban	0. no		0. no			0. no

2. oly read	5. house w	1. amhara	2. urban	0. no	0. non iodi:	1. yes	44	3	1. yes
2. oly read	5. house w	1. amhara	1. rural	0. no	1. iodized	1. yes	39	2.5	0. no
5. college ‹	5. house w	1. amhara	2. urban	0. no	1. iodized	0. no	38.8		0. no
3. grade 1-	5. house w	1. amhara	1. rural	1. yes	1. iodized	1. yes	38.5	8	0. no
2. oly read	5. house w	1. amhara	2. urban	0. no	0. non iodi:	1. yes	38.5	10	0. no
4. grade 9-	5. house w	1. amhara	1. rural	0. no	0. non iodi:	1. yes	38.5	2	0. no
5. college ‹	1. goverme	1. amhara	2. urban	0. no	1. iodized	0. no	38.5		0. no
3. grade 1-	5. house w	1. amhara	2. urban	0. no	1. iodized	1. yes	38.5	3	0. no
3. grade 1-	5. house w	1. amhara	2. urban	0. no	1. iodized	1. yes	38.5		0. no
3. grade 1-	2. merchat	1. amhara	2. urban	0. no	1. iodized	1. yes	38.5		0. no
2. oly read	5. house w	1. amhara	1. rural	0. no	0. non iodi:	0. no	38.5	2	0. no
3. grade 1-	2. merchat	2. oromo	2. urban	0. no	1. iodized	1. yes	39	2.5	0. no
2. oly read	5. house w	1. amhara	2. urban	0. no	0. non iodi:	1. yes	44	3	1. yes
2. oly read	5. house w	1. amhara	1. rural	0. no	1. iodized	1. yes	39	2.5	0. no
5. college ‹	1. goverme	1. amhara	2. urban	0. no	1. iodized	1. yes	40	0	0. no
4. grade 9-	5. house w	1. amhara	2. urban	0. no	1. iodized	0. no	38.5		0. no
5. college ‹	2. merchat	1. amhara	2. urban	0. no	1. iodized	1. yes	38.8	2	0. no
3. grade 1-	5. house w	1. amhara	1. rural	1. yes	1. iodized	1. yes	38.5	8	0. no
5. college ‹	1. goverme	1. amhara	2. urban	0. no	1. iodized	1. yes	38.8	0.6	0. no
3. grade 1-	5. house w	1. amhara	2. urban	0. no	1. iodized	1. yes	38.5	3	0. no
4. grade 9-	5. house w	1. amhara	2. urban	0. no	1. iodized	0. no	38.5		0. no
3. grade 1-	5. house w	1. amhara	2. urban	0. no	1. iodized	1. yes	38.5		0. no
5. college ‹	2. merchat	1. amhara	2. urban	0. no	1. iodized	1. yes	38.8	2	0. no
3. grade 1-	2. merchat	1. amhara	2. urban	0. no		0. no	38.5	2	0. no
5. college ‹	1. goverme	1. amhara	2. urban	0. no	1. iodized	1. yes	38.5	2.75	0. no
3. grade 1-	5. house w	1. amhara	1. rural	1. yes	1. iodized	1. yes	38.5	8	0. no
5. college ‹	5. house w	1. amhara	2. urban	0. no	1. iodized	1. yes	42	0	0. no
5. college ‹	1. goverme	1. amhara	2. urban	0. no	1. iodized	1. yes	38.8	0.6	0. no
4. grade 9-	2. merchat	1. amhara	2. urban	0. no	1. iodized	0. no	38.6	3.5	0. no
3. grade 1-	2. merchat	2. oromo	2. urban	0. no	1. iodized	1. yes	39	2.5	0. no
2. oly read	5. house w	1. amhara	2. urban	0. no	0. non iodi:	1. yes	44	3	1. yes
3. grade 1-	2. merchat	1. amhara	2. urban	0. no		0. no	38.5	2	0. no
5. college ‹	5. house w	1. amhara	2. urban	0. no	1. iodized	0. no	38.8		0. no
3. grade 1-	5. house w	1. amhara	1. rural	0. no	0. non iodi:	1. yes	40	10	0. no
2. oly read	5. house w	1. amhara	2. urban	0. no	0. non iodi:	1. yes	38.5	10	0. no
5. college ‹	1. goverme	1. amhara	2. urban	0. no	1. iodized	1. yes	40	0	0. no
5. college ‹	1. goverme	1. amhara	2. urban	0. no	1. iodized	1. yes	38.8	0.6	0. no
4. grade 9-	5. house w	1. amhara	2. urban	0. no	1. iodized	1. yes	35	13	1. yes
4. grade 9-	5. house w	1. amhara	2. urban	0. no	1. iodized	0. no	38.5		0. no
3. grade 1-	2. merchat	1. amhara	2. urban	0. no	1. iodized	1. yes	38.5		0. no
4. grade 9-	5. house w	1. amhara	2. urban	0. no	1. iodized	0. no	38.5		0. no
4. grade 9-	5. house w	1. amhara	2. urban	0. no	1. iodized	0. no	38.5		0. no
3. grade 1-	5. house w	1. amhara	2. urban	0. no	1. iodized	1. yes	38.5	3	0. no
5. college ‹	1. goverme	1. amhara	2. urban	0. no	1. iodized	0. no	40	2.6	0. no
5. college ‹	5. house w	1. amhara	2. urban	1. yes	1. iodized	1. yes	38.5	3	1. yes
4. grade 9-	5. house w	1. amhara	2. urban	0. no	1. iodized	0. no	38.5		0. no
3. grade 1-	3. private ‹	4. others	1. rural	1. yes	1. iodized	0. no	41	40	0. no
3. grade 1-	5. house w	1. amhara	2. urban	0. no	1. iodized	1. yes	38.5		0. no
4. grade 9-	5. house w	1. amhara	1. rural	0. no	1. iodized	1. yes	42.8		0. no
2. oly read	5. house w	1. amhara	1. rural	0. no	1. iodized	1. yes	39	2.5	0. no
5. college ‹	1. goverme	1. amhara	2. urban	0. no	1. iodized	1. yes	40	0	0. no
4. grade 9-	5. house w	2. oromo	2. urban	0. no	1. iodized	1. yes	41		0. no

4. grade 9-	5. house w	1. amhara	2. urban	0. no	1. iodized	1. yes	38.5	8	0. no

0. no	1. yes	1. yes	1. yes	1. yes					
1. yes									
0. no	1. yes	1. yes							
0. no									
0. no									
0. no									
0. no									
1. yes	1. yes	1. yes	1. yes	1. yes	1. yes	1. yes	1. yes	1. yes	1. yes
0. no									
0. no									
0. no									
0. no									
0. no									
1. yes	1. yes	1. yes	1. yes	1. yes					
1. yes	0. no	0. no							
0. no									
1. yes									
0. no									
0. no									
1. yes	1. yes	0. no	0. no	0. no					
1. yes									
0. no	1. yes	1. yes	1. yes	1. yes					
1. yes					1. yes	1. yes	1. yes	1. yes	1. yes
0. no	1. yes	1. yes	1. yes	1. yes					
1. yes									
0. no					1. yes	1. yes	1. yes	1. yes	1. yes
1. yes									
0. no					0. no	1. yes	1. yes	1. yes	1. yes
0. no									
0. no									
0. no				0. no	1. yes				
0. no	0. no	1. yes	1. yes						
0. no	1. yes	1. yes	1. yes	1. yes					
0. no					1. yes	1. yes	1. yes	1. yes	1. yes
1. yes									
0. no									
0. no									
0. no									
0. no	1. yes	1. yes							
0. no									
0. no									
1. yes									
1. yes					1. yes	1. yes	1. yes	1. yes	1. yes
0. no					1. yes	1. yes	0. no	0. no	0. no
0. no									
	1. yes	1. yes	1. yes	1. yes					
0. no									
0. no									
0. no									
1. yes	1. yes	1. yes	1. yes	1. yes					

0. no	1. yes	1. yes	1. yes	1. yes					
0. no	1. yes	0. no	1. yes						
0. no									
0. no									
	0. no	0. no	0. no	0. no					
0. no									
0. no	0. no								
	1. yes	1. yes	1. yes	1. yes					
0. no	1. yes	1. yes	0. no	0. no					
0. no									
0. no									
0. no	1. yes	1. yes	1. yes	1. yes					
0. no									
0. no					1. yes	1. yes	1. yes	1. yes	1. yes
1. yes									
0. no	1. yes	1. yes	1. yes	1. yes					
0. no									
0. no									
0. no									
0. no									
0. no									
0. no									
1. yes	1. yes	1. yes	1. yes	1. yes					
1. yes	1. yes	0. no	0. no	0. no					
1. yes									
1. yes					1. yes	1. yes	1. yes	1. yes	1. yes
0. no					0. no	1. yes	1. yes	1. yes	1. yes
0. no									
0. no				0. no	1. yes				
0. no	1. yes	1. yes	1. yes	1. yes					
1. yes									
0. no									
0. no	0. no								
0. no	1. yes	1. yes	1. yes	1. yes					
0. no									
0. no	1. yes	1. yes							
0. no									
1. yes	0. no	0. no							
1. yes									
0. no									
0. no									
0. no	1. yes	1. yes	1. yes	1. yes					
1. yes					1. yes	1. yes	1. yes	1. yes	1. yes
0. no	1. yes	1. yes	1. yes	1. yes					
0. no				0. no	1. yes				
1. yes									
1. yes					1. yes	1. yes	1. yes	1. yes	1. yes
0. no									
0. no	1. yes	1. yes	1. yes	1. yes					
0. no									

0. no									
0. no									
0. no	1. yes	1. yes	1. yes	1. yes					
0. no									
0. no	1. yes	1. yes	1. yes	1. yes					
1. yes									
0. no	1. yes	1. yes							
0. no									
0. no									
0. no									
0. no									
0. no									
0. no									
1. yes	1. yes	1. yes	1. yes	1. yes					
1. yes	0. no	0. no							
0. no									
1. yes									
1. yes	1. yes	0. no	0. no	0. no					
1. yes					1. yes	1. yes	1. yes	1. yes	1. yes
0. no	1. yes	1. yes	1. yes	1. yes					
1. yes									
0. no									
0. no				0. no	1. yes				
0. no	0. no	1. yes	1. yes						
0. no					1. yes	1. yes	1. yes	1. yes	1. yes
0. no									
0. no									
0. no									
0. no									
1. yes									
1. yes					1. yes	1. yes	1. yes	1. yes	1. yes
0. no									
1. yes	1. yes	1. yes	1. yes	1. yes					
0. no	1. yes	1. yes	1. yes	1. yes					
0. no									
0. no	1. yes	1. yes	0. no	0. no					
0. no									
0. no									
0. no					1. yes	1. yes	1. yes	1. yes	1. yes
1. yes									
0. no									
0. no									
0. no									
0. no									
1. yes									
0. no	1. yes	1. yes	1. yes	1. yes					
0. no	1. yes	1. yes	1. yes	1. yes					
0. no									
0. no	1. yes	1. yes							
1. yes	0. no	0. no							

1. yes									
0. no									
0. no									
0. no									
0. no									
0. no									
1. yes	1. yes	1. yes	1. yes	1. yes					
1. yes	1. yes	0. no	0. no	0. no					
1. yes									
1. yes									
1. yes									
1. yes					1. yes	1. yes	1. yes	1. yes	1. yes
0. no					1. yes	1. yes	0. no	0. no	0. no
	1. yes	1. yes	1. yes	1. yes					
0. no									
0. no									
0. no	1. yes	1. yes	1. yes	1. yes					
0. no									
0. no									
	0. no	0. no	0. no	0. no					
0. no									
0. no									
0. no									
0. no	1. yes	1. yes	1. yes	1. yes					
0. no									
0. no									
1. yes					1. yes	1. yes	1. yes	1. yes	1. yes
0. no									
0. no	1. yes	1. yes	1. yes	1. yes					
1. yes									
0. no	1. yes	1. yes	1. yes	1. yes					
0. no									
0. no									
0. no				0. no	1. yes				
0. no									
0. no	1. yes	1. yes							
0. no									
0. no									
0. no									
0. no									
0. no	0. no	1. yes	1. yes						
0. no									
1. yes									
1. yes					1. yes	1. yes	1. yes	1. yes	1. yes
1. yes	1. yes	1. yes	1. yes	1. yes					
0. no									
0. no	1. yes	1. yes	0. no	0. no					
1. yes									
0. no									
0. no									

0. no									
1. yes	1. yes	1. yes	1. yes	1. yes					
0. no									
0. no	1. yes	1. yes	1. yes	1. yes					
1. yes									
0. no	1. yes	1. yes	1. yes	1. yes					
0. no									
0. no									
1. yes	1. yes	1. yes	1. yes	1. yes					
0. no	1. yes	1. yes	1. yes	1. yes					
0. no									
0. no									
0. no									
0. no									
1. yes	1. yes	1. yes	1. yes	1. yes					
1. yes					1. yes	1. yes	1. yes	1. yes	1. yes
1. yes									
0. no									
0. no	0. no								
0. no									
0. no	1. yes	1. yes	1. yes	1. yes					
1. yes					1. yes	1. yes	1. yes	1. yes	1. yes
0. no									
0. no	1. yes	1. yes	1. yes	1. yes					
1. yes									
0. no									
0. no									
0. no	0. no	1. yes	1. yes						
0. no									
0. no									
1. yes									
1. yes					1. yes	1. yes	1. yes	1. yes	1. yes
0. no									
0. no	1. yes	1. yes							
1. yes	0. no	0. no							
0. no									
0. no									
1. yes	1. yes	1. yes	1. yes	1. yes					
1. yes	1. yes	0. no	0. no	0. no					
1. yes									
1. yes									
0. no									
0. no									
	0. no	0. no	0. no	0. no					
0. no									
0. no									
0. no									
0. no									
0. no									

1. yes									
1. yes									
0. no	1. yes	0. no	1. yes						
0. no									
0. no									
0. no									
0. no									
0. no	0. no								
0. no									
0. no				0. no	1. yes				
0. no									
0. no									
0. no	1. yes	1. yes	1. yes	1. yes					
0. no	1. yes	1. yes							
1. yes	0. no	0. no							
0. no									
1. yes	1. yes	0. no	0. no	0. no					
1. yes									
1. yes									
0. no									
1. yes									
1. yes	1. yes	1. yes	1. yes	1. yes					
1. yes									
1. yes									
0. no	1. yes	1. yes	1. yes	1. yes					
0. no									
0. no									
0. no									
1. yes					1. yes	1. yes	1. yes	1. yes	1. yes
0. no	1. yes	1. yes	1. yes	1. yes					
1. yes									
0. no									
0. no	0. no	1. yes	1. yes						
1. yes					1. yes	1. yes	1. yes	1. yes	1. yes
0. no									
0. no									
0. no	1. yes	1. yes	1. yes	1. yes					
0. no									
1. yes	1. yes	1. yes	1. yes	1. yes					
1. yes					1. yes	1. yes	1. yes	1. yes	1. yes
1. yes									
0. no									
0. no	0. no								
0. no	0. no	1. yes	1. yes						
1. yes	1. yes	1. yes	1. yes	1. yes					
1. yes	1. yes	0. no	0. no	0. no					
0. no	1. yes	1. yes	1. yes	1. yes					
0. no									
0. no									
0. no									

1. yes	1. yes	1. yes	1. yes	1. yes					
1. yes	0. no	0. no							
0. no									
1. yes	1. yes	0. no	0. no	0. no					
1. yes									
1. yes					1. yes	1. yes	1. yes	1. yes	1. yes
1. yes									
0. no					0. no	1. yes	1. yes	1. yes	1. yes
0. no				0. no	1. yes				
0. no									
1. yes					1. yes	1. yes	1. yes	1. yes	1. yes
0. no									
	1. yes	1. yes	1. yes	1. yes					
0. no									
	0. no	0. no	0. no	0. no					
0. no									
0. no	0. no								
0. no									
0. no					1. yes	1. yes	1. yes	1. yes	1. yes
0. no									
0. no									
0. no									
1. yes	1. yes	0. no	0. no	0. no					
1. yes									
1. yes					1. yes	1. yes	1. yes	1. yes	1. yes
0. no									
0. no	0. no								
0. no									
0. no	1. yes	1. yes							
0. no									
0. no									
1. yes					1. yes	1. yes	1. yes	1. yes	1. yes
0. no	1. yes	1. yes	1. yes	1. yes					
0. no									
0. no	1. yes	1. yes	1. yes	1. yes					
1. yes									
0. no	1. yes	1. yes							
0. no									
0. no									
0. no									
0. no									
0. no									
1. yes	0. no	0. no							
1. yes	1. yes	0. no	0. no	0. no					
0. no									
0. no									
0. no	1. yes	1. yes	1. yes	1. yes					

0. no									
0. no									
0. no									
0. no									
1. yes									
0. no									
0. no									
0. no									
1. yes	1. yes	1. yes	1. yes	1. yes					
1. yes									
1. yes					1. yes	1. yes	1. yes	1. yes	1. yes
0. no									
0. no									
0. no									
0. no									
1. yes									
0. no									
0. no									
0. no	1. yes	1. yes							
0. no									
1. yes									
1. yes									
0. no									
0. no									
0. no									
0. no									
0. no	0. no	1. yes	1. yes						
0. no	1. yes	1. yes							
1. yes	1. yes	0. no	0. no	0. no					
0. no									
0. no									
0. no									
0. no									
0. no									
1. yes									
0. no									
0. no	1. yes	1. yes							
1. yes	0. no	0. no							
1. yes									
1. yes									
1. yes									
1. yes									
0. no									
0. no									
0. no	1. yes	1. yes	1. yes	1. yes					
1. yes									
0. no	0. no								
1. yes	1. yes	1. yes	1. yes	1. yes					
0. no	1. yes	1. yes	1. yes	1. yes					
0. no									
0. no									
0. no	1. yes	1. yes	1. yes	1. yes					

0. no

					0. no	0. no	1. yes	0. no	
1. yes	1. yes	1. yes							
					1. yes	1. yes			
					1. yes	1. yes	1. yes	0. no	1. yes
							1. yes	1. yes	1. yes
							1. yes	1. yes	1. yes
1. yes	1. yes								
1. yes	1. yes	1. yes							
1. yes									
						1. yes	1. yes	1. yes	1. yes
						1. yes	1. yes	1. yes	
						1. yes	1. yes	1. yes	1. yes
						1. yes	0. no	0. no	0. no
1. yes	0. no								
1. yes									
					1. yes	1. yes	1. yes	1. yes	1. yes

				0. no	1. yes	1. yes	0. no
			1. yes	1. yes	1. yes	1. yes	1. yes
			1. yes	1. yes	1. yes	0. no	0. no
				1. yes	1. yes	1. yes	0. no
				1. yes	1. yes	1. yes	1. yes
1. yes	1. yes	1. yes					
			0. no	0. no	0. no	1. yes	1. yes
			1. yes	1. yes			
1. yes	1. yes						
1. yes							
				1. yes	1. yes	1. yes	
			1. yes	1. yes	1. yes	1. yes	1. yes
				1. yes	1. yes	1. yes	1. yes
					1. yes	1. yes	1. yes
					1. yes	1. yes	1. yes
1. yes	1. yes						
1. yes	0. no						
			1. yes	1. yes	1. yes	0. no	0. no

				1. yes	1. yes	1. yes	1. yes
			0. no	0. no	1. yes	0. no	
			1. yes	1. yes			
1. yes	1. yes						
				1. yes	1. yes	1. yes	
				1. yes	1. yes	1. yes	1. yes
				1. yes	0. no	0. no	0. no
1. yes	0. no						
			1. yes	1. yes	1. yes	1. yes	1. yes
				1. yes	1. yes	1. yes	0. no
				1. yes	1. yes	1. yes	1. yes
1. yes	1. yes	1. yes					
			0. no	0. no	0. no	1. yes	1. yes
			1. yes	1. yes			
				1. yes	1. yes	1. yes	1. yes

				1. yes	1. yes	1. yes
		0. no	0. no	1. yes	0. no	
		1. yes	1. yes			
		1. yes	1. yes	1. yes	0. no	1. yes
1. yes	0. no					
1. yes						
		1. yes	1. yes	1. yes	1. yes	1. yes
			0. no	1. yes	1. yes	0. no
		1. yes	1. yes	1. yes	1. yes	1. yes
			1. yes	1. yes	1. yes	0. no
			1. yes	1. yes	1. yes	1. yes
		1. yes	1. yes			
1. yes	1. yes					
			1. yes	1. yes	1. yes	
			1. yes	1. yes	1. yes	1. yes
		1. yes	1. yes			
			1. yes	1. yes	1. yes	
			1. yes	1. yes	1. yes	1. yes
1. yes	0. no					
		1. yes	1. yes	1. yes	1. yes	1. yes
		0. no	0. no	0. no	1. yes	1. yes

			1. yes	1. yes	1. yes	1. yes
		1. yes	1. yes	1. yes	1. yes	1. yes
1. yes	1. yes					
		1. yes	1. yes	1. yes	1. yes	1. yes
				1. yes	1. yes	1. yes
1. yes	0. no					
			1. yes	1. yes	1. yes	
1. yes	0. no					
			1. yes	1. yes	1. yes	1. yes
		1. yes	1. yes			
		1. yes	1. yes	1. yes	0. no	1. yes
			0. no	1. yes	1. yes	0. no
			1. yes	1. yes	1. yes	0. no
			1. yes	1. yes	1. yes	1. yes

			1. yes	1. yes	1. yes	1. yes
		1. yes	1. yes			
			1. yes	1. yes	1. yes	
			1. yes	1. yes	1. yes	1. yes
			1. yes	1. yes	1. yes	0. no
			1. yes	1. yes	1. yes	0. no
			1. yes	1. yes	1. yes	1. yes
1. yes	1. yes					
		1. yes	1. yes			
1. yes	0. no					
		1. yes	1. yes	1. yes	1. yes	1. yes
		1. yes	1. yes	1. yes	1. yes	1. yes
1. yes	1. yes					
		1. yes	1. yes	1. yes	1. yes	1. yes

					1. yes	1. yes	1. yes
1. yes	1. yes						
1. yes							
1. yes	0. no						
				0. no	1. yes	1. yes	0. no
			1. yes	1. yes	1. yes	1. yes	1. yes
			1. yes	1. yes	1. yes	0. no	0. no
1. yes	1. yes	1. yes					
			1. yes	1. yes			
1. yes	1. yes						
			1. yes	1. yes	1. yes	1. yes	1. yes
				1. yes	1. yes	1. yes	1. yes
					1. yes	1. yes	1. yes
					1. yes	1. yes	1. yes
1. yes	0. no						
			0. no	0. no	1. yes	0. no	

			1. yes	1. yes	1. yes	0. no
			1. yes	1. yes	1. yes	1. yes
				1. yes	1. yes	1. yes
		0. no	0. no	1. yes	0. no	
		1. yes	1. yes			
1. yes	0. no					
			0. no	1. yes	1. yes	0. no
			1. yes	1. yes	1. yes	0. no
			1. yes	1. yes	1. yes	1. yes
			1. yes	1. yes	1. yes	
		1. yes	1. yes			
		0. no	0. no	0. no	1. yes	1. yes
			1. yes	1. yes	1. yes	1. yes
		1. yes	1. yes	1. yes	1. yes	1. yes
			0. no	1. yes	1. yes	0. no
			1. yes	1. yes	1. yes	0. no
			1. yes	1. yes	1. yes	1. yes
			1. yes	1. yes	1. yes	
		1. yes	1. yes			
		1. yes	1. yes	1. yes	1. yes	1. yes
			1. yes	1. yes	1. yes	1. yes
			1. yes	1. yes	1. yes	

									less tha 12
		1. yes	1. yes	1. yes					36-47 motl
									less tha 12
		1. yes	1. yes	1. yes	1. yes				48-59 motl
						1. yes	1. yes	1. yes	48-59 motl
						1. yes	1. yes	1. yes	48-59 motl
									24-35 motl
									12-23 motl
									24-35 motl
						1. yes	1. yes	1. yes	48-59 motl
		1. yes	1. yes	1. yes	1. yes				36-47 motl
						1. yes	1. yes	1. yes	48-59 motl
1. yes									24-35 motl
									less tha 12
									less tha 12
		1. yes	1. yes	1. yes	1. yes				36-47 motl
		0. no	0. no	0. no	0. no				36-47 motl
1. yes	1. yes								24-35 motl
1. yes	1. yes								24-35 motl
									less tha 12
						1. yes	1. yes	1. yes	48-59 motl
									36-47 motl
									12-23 motl
									12-23 motl
									less tha 12
						1. yes	1. yes	1. yes	48-59 motl
									12-23 motl
						0. no	0. no	0. no	48-59 motl
									12-23 motl
1. yes	1. yes								24-35 motl
									24-35 motl
									12-23 motl
									12-23 motl
									less tha 12
									12-23 motl
						1. yes	1. yes	1. yes	48-59 motl
						1. yes	1. yes	1. yes	48-59 motl
		1. yes	1. yes	1. yes	1. yes				36-47 motl
									24-35 motl
									less tha 12
		1. yes	1. yes	1. yes	1. yes				36-47 motl
0. no	0. no								24-35 motl
		1. yes	1. yes	1. yes	1. yes				36-47 motl
									12-23 motl
									12-23 motl
		1. yes	0. no	0. no	0. no				36-47 motl
									less tha 12
						1. yes	1. yes	1. yes	48-59 motl
						1. yes	1. yes	1. yes	48-59 motl
1. yes	1. yes								24-35 motl
						1. yes	1. yes	1. yes	48-59 motl
									less tha 12

									less tha 12
									less tha 12
0. no	0. no								36-47 motl
									36-47 motl
		1. yes	1. yes	1. yes	1. yes				36-47 motl
									less tha 12
1. yes	1. yes								24-35 motl
						1. yes	1. yes	1. yes	48-59 motl
									less tha 12
									less tha 12
									24-35 motl
0. no	0. no								24-35 motl
									less tha 12
1. yes	1. yes								24-35 motl
									12-23 motl
1. yes	1. yes								24-35 motl
									less tha 12
		1. yes	1. yes	1. yes	1. yes				48-59 motl
						1. yes	1. yes	1. yes	48-59 motl
									24-35 motl
						1. yes	1. yes	1. yes	48-59 motl
		1. yes	1. yes	1. yes	1. yes				36-47 motl
						1. yes	1. yes	1. yes	48-59 motl
									less tha 12
									less tha 12
						1. yes	1. yes	1. yes	48-59 motl
									12-23 motl
									12-23 motl
									24-35 motl
									12-23 motl
									less tha 12
		1. yes	1. yes	1. yes	1. yes				36-47 motl
1. yes	1. yes								24-35 motl
						1. yes	1. yes	1. yes	48-59 motl
									less tha 12
1. yes	1. yes								24-35 motl
									less tha 12
						1. yes	1. yes	1. yes	48-59 motl
									less tha 12
		0. no	0. no	0. no	0. no				36-47 motl
1. yes	1. yes								24-35 motl
1. yes	1. yes								24-35 motl
									36-47 motl
									12-23 motl
									12-23 motl
									less tha 12
									12-23 motl
		1. yes	1. yes	1. yes	1. yes				36-47 motl
									12-23 motl
						1. yes	1. yes	1. yes	48-59 motl
									less tha 12
									24-35 motl

		1. yes	1. yes	1. yes	1. yes				36-47 motl
						1. yes	1. yes	1. yes	48-59 motl
									less tha 12
1. yes	1. yes								24-35 motl
									less tha 12
		1. yes	1. yes	1. yes					36-47 motl
									less tha 12
		1. yes	1. yes	1. yes	1. yes				48-59 motl
						1. yes	1. yes	1. yes	48-59 motl
									24-35 motl
									24-35 motl
						1. yes	1. yes	1. yes	48-59 motl
						1. yes	1. yes	1. yes	48-59 motl
									less tha 12
									less tha 12
		1. yes	1. yes	1. yes	1. yes				36-47 motl
		0. no	0. no	0. no	0. no				36-47 motl
									less tha 12
									12-23 motl
									less tha 12
						0. no	0. no	0. no	48-59 motl
									24-35 motl
									12-23 motl
									12-23 motl
									12-23 motl
						1. yes	1. yes	1. yes	48-59 motl
									24-35 motl
		1. yes	1. yes	1. yes	1. yes				36-47 motl
0. no	0. no								24-35 motl
		1. yes	1. yes	1. yes	1. yes				36-47 motl
									12-23 motl
		1. yes	0. no	0. no	0. no				36-47 motl
						1. yes	1. yes	1. yes	48-59 motl
									less tha 12
									less tha 12
									36-47 motl
1. yes	1. yes								24-35 motl
									less tha 12
0. no	0. no								24-35 motl
1. yes	1. yes								24-35 motl
									12-23 motl
1. yes	1. yes								24-35 motl
									24-35 motl
						1. yes	1. yes	1. yes	48-59 motl
		1. yes	1. yes	1. yes	1. yes				36-47 motl
						1. yes	1. yes	1. yes	48-59 motl
						1. yes	1. yes	1. yes	48-59 motl
									less tha 12
									less tha 12
1. yes	1. yes								24-35 motl
									less tha 12
									less tha 12

		0. no	0. no	0. no	0. no				36-47 motl
1. yes	1. yes								24-35 motl
						1. yes	1. yes	1. yes	48-59 motl
									24-35 motl
									24-35 motl
1. yes									24-35 motl
									less tha 12
									less tha 12
									36-47 motl
						1. yes	1. yes	1. yes	48-59 motl
						1. yes	1. yes	1. yes	48-59 motl
		1. yes	1. yes	1. yes	1. yes				36-47 motl
									12-23 motl
									12-23 motl
									less tha 12
1. yes	1. yes								24-35 motl
						1. yes	1. yes	1. yes	48-59 motl
									less tha 12
0. no	0. no								36-47 motl
		1. yes	1. yes	1. yes	1. yes				36-47 motl
									less tha 12
1. yes	1. yes								24-35 motl
0. no	0. no								24-35 motl
1. yes	1. yes								24-35 motl
									less tha 12
		1. yes	1. yes	1. yes	1. yes				48-59 motl
									24-35 motl
									12-23 motl
									24-35 motl
									less tha 12
		1. yes	1. yes	1. yes	1. yes				36-47 motl
									less tha 12
1. yes	1. yes								24-35 motl
						1. yes	1. yes	1. yes	48-59 motl
									12-23 motl
		1. yes	1. yes	1. yes	1. yes				36-47 motl
									less tha 12
						1. yes	1. yes	1. yes	48-59 motl
									24-35 motl
		1. yes	1. yes	1. yes	1. yes				36-47 motl
									24-35 motl
									12-23 motl
									24-35 motl
		1. yes	1. yes	1. yes	1. yes				36-47 motl
									12-23 motl
									less tha 12
									36-47 motl
1. yes	1. yes								24-35 motl
									less tha 12
1. yes	1. yes								24-35 motl
		1. yes	1. yes	1. yes	1. yes				36-47 motl
						1. yes	1. yes	1. yes	48-59 motl

						1. yes	1. yes	1. yes	48-59 motl
									less tha 12
		1. yes	1. yes	1. yes	1. yes				36-47 motl
									less tha 12
						0. no	0. no	0. no	48-59 motl
									less tha 12
									24-35 motl
						1. yes	1. yes	1. yes	48-59 motl
1. yes	1. yes								24-35 motl
									less tha 12
									36-47 motl
									less tha 12
						1. yes	1. yes	1. yes	48-59 motl
						1. yes	1. yes	1. yes	48-59 motl
		1. yes	1. yes	1. yes	1. yes				36-47 motl
						1. yes	1. yes	1. yes	48-59 motl
									less tha 12
									12-23 motl
		1. yes	1. yes	1. yes	1. yes				36-47 motl
1. yes	1. yes								24-35 motl
						1. yes	1. yes	1. yes	48-59 motl
1. yes	1. yes								24-35 motl
									less tha 12
									12-23 motl
		1. yes	1. yes	1. yes	1. yes				36-47 motl
									less tha 12
		1. yes	1. yes	1. yes					36-47 motl
		1. yes	1. yes	1. yes	1. yes				36-47 motl
									24-35 motl
									12-23 motl
						1. yes	1. yes	1. yes	48-59 motl
		1. yes	1. yes	1. yes	1. yes				36-47 motl
		1. yes	1. yes	1. yes	1. yes				36-47 motl
									12-23 motl
						1. yes	1. yes	1. yes	48-59 motl
1. yes	1. yes								24-35 motl
									less tha 12
									less tha 12
									24-35 motl
1. yes									24-35 motl
									less tha 12
									less tha 12
						1. yes	1. yes	1. yes	48-59 motl
		1. yes	1. yes	1. yes	1. yes				36-47 motl
0. no	0. no								36-47 motl
		1. yes	1. yes	1. yes	1. yes				36-47 motl
									less tha 12
0. no	0. no								24-35 motl
						1. yes	1. yes	1. yes	48-59 motl
									24-35 motl
						1. yes	1. yes	1. yes	48-59 motl
		1. yes	1. yes	1. yes	1. yes				36-47 motl

		0. no	0. no	0. no	0. no				36-47 motl
									36-47 motl
						1. yes	1. yes	1. yes	48-59 motl
									less tha 12
1. yes	1. yes								24-35 motl
		1. yes	1. yes	1. yes	1. yes				48-59 motl
									24-35 motl
									24-35 motl
						1. yes	1. yes	1. yes	48-59 motl
1. yes	1. yes								24-35 motl
									12-23 motl
						1. yes	1. yes	1. yes	48-59 motl
		1. yes	1. yes	1. yes	1. yes				36-47 motl
									less tha 12
									less tha 12
									less tha 12
		1. yes	1. yes	1. yes	1. yes				36-47 motl
									less tha 12
						0. no	0. no	0. no	48-59 motl
		1. yes	1. yes	1. yes	1. yes				36-47 motl
0. no	0. no								24-35 motl
						1. yes	1. yes	1. yes	48-59 motl
									less tha 12
									36-47 motl
						1. yes	1. yes	1. yes	48-59 motl
		1. yes	1. yes	1. yes	1. yes				36-47 motl
									less tha 12
0. no	0. no								24-35 motl
1. yes	1. yes								24-35 motl
		1. yes	1. yes	1. yes	1. yes				48-59 motl
									12-23 motl
									less tha 12
		1. yes	1. yes	1. yes	1. yes				36-47 motl
									24-35 motl
									12-23 motl
									12-23 motl
1. yes	1. yes								24-35 motl
1. yes	1. yes								24-35 motl
									less tha 12
						1. yes	1. yes	1. yes	48-59 motl
									less tha 12
									12-23 motl
		1. yes	1. yes	1. yes	1. yes				36-47 motl
1. yes	1. yes								24-35 motl
						1. yes	1. yes	1. yes	48-59 motl
									12-23 motl
									less tha 12
									less tha 12
									less tha 12
		1. yes	1. yes	1. yes	1. yes				48-59 motl
						1. yes	1. yes	1. yes	48-59 motl
						1. yes	1. yes	1. yes	48-59 motl

									less tha 12
									less tha 12
1. yes	1. yes								24-35 motl
									less tha 12
						1. yes	1. yes	1. yes	48-59 motl
									36-47 motl
									12-23 motl
						0. no	0. no	0. no	48-59 motl
									12-23 motl
									12-23 motl
		1. yes	1. yes	1. yes	1. yes				36-47 motl
									12-23 motl
		1. yes	0. no	0. no	0. no				36-47 motl
									less tha 12
0. no	0. no								36-47 motl
									36-47 motl
									less tha 12
1. yes	1. yes								24-35 motl
						1. yes	1. yes	1. yes	48-59 motl
									24-35 motl
									12-23 motl
		1. yes	1. yes	1. yes	1. yes				48-59 motl
									24-35 motl
						1. yes	1. yes	1. yes	48-59 motl
									less tha 12
						1. yes	1. yes	1. yes	48-59 motl
									12-23 motl
1. yes	1. yes								24-35 motl
						1. yes	1. yes	1. yes	48-59 motl
1. yes	1. yes								24-35 motl
									less tha 12
1. yes	1. yes								24-35 motl
1. yes	1. yes								24-35 motl
									36-47 motl
									12-23 motl
									less tha 12
		1. yes	1. yes	1. yes	1. yes				36-47 motl
									less tha 12
		1. yes	1. yes	1. yes					36-47 motl
									less tha 12
		1. yes	1. yes	1. yes	1. yes				48-59 motl
						1. yes	1. yes	1. yes	48-59 motl
									24-35 motl
						1. yes	1. yes	1. yes	48-59 motl
						1. yes	1. yes	1. yes	48-59 motl
									less tha 12
									less tha 12
		1. yes	1. yes	1. yes	1. yes				36-47 motl
		1. yes	0. no	0. no	0. no				36-47 motl
						1. yes	1. yes	1. yes	48-59 motl
									less tha 12
									36-47 motl

0. no	0. no								24-35 motl
1. yes	1. yes								24-35 motl
						1. yes	1. yes	1. yes	48-59 motl
		1. yes	1. yes	1. yes	1. yes				36-47 motl
		0. no	0. no	0. no	0. no				36-47 motl
1. yes	1. yes								24-35 motl
									24-35 motl
									24-35 motl
									less tha 12
						1. yes	1. yes	1. yes	48-59 motl
									12-23 motl
0. no	0. no								36-47 motl
0. no	0. no								24-35 motl
1. yes	1. yes								24-35 motl
									24-35 motl
		1. yes	1. yes	1. yes	1. yes				36-47 motl
						1. yes	1. yes	1. yes	48-59 motl
		1. yes	1. yes	1. yes	1. yes				36-47 motl
									less tha 12
									24-35 motl
		1. yes	1. yes	1. yes	1. yes				36-47 motl
1. yes	1. yes								24-35 motl
						1. yes	1. yes	1. yes	48-59 motl
									24-35 motl
1. yes	1. yes								24-35 motl
		1. yes	1. yes	1. yes	1. yes				36-47 motl
									12-23 motl
									less tha 12
									less tha 12
0. no	0. no								36-47 motl
0. no	0. no								24-35 motl
									24-35 motl
						1. yes	1. yes	1. yes	48-59 motl
		1. yes	1. yes	1. yes	1. yes				36-47 motl
		0. no	0. no	0. no	0. no				36-47 motl
									24-35 motl
									less tha 12
									less tha 12
		1. yes	1. yes	1. yes	1. yes				36-47 motl
						1. yes	1. yes	1. yes	48-59 motl
		1. yes	1. yes	1. yes	1. yes				36-47 motl
		1. yes	1. yes	1. yes	1. yes				36-47 motl
									24-35 motl
1. yes	1. yes								24-35 motl
									less tha 12
		1. yes	1. yes	1. yes	1. yes				36-47 motl
						1. yes	1. yes	1. yes	48-59 motl
									less tha 12
									less tha 12
1. yes	1. yes								24-35 motl
									24-35 motl
									less tha 12

1. yes	1. yes	1. yes	1. yes	48-59 motl

	4	1					1			
	3				1					1
	2	1					1			
	4									
	3					1				
	3					1				
	1			0					0.25	
	12		1					1		
	2			1					1	
	3					1				
	4				1					1
	3					1				
	5			1					0.833333	
	4	1					1			
	0	0					0			
	4				1					1
	0				0					0
	5			1					1	
	5			1					1	
	1	0					0.25			
	3					1				
ns										
	4									
	7		1					1		
	4	1					1			
	3					1				
	8		1					1		
	0					0				
	5		1					0.833333		
	6			1					1	
	3			1					1	
	1		1					1		
	2									
	4	1					1			
	5		1					1		
	3					1				
	3					1				
	4				1					1
	4			1					1	
	2	1					1			
	4				1					1
	1			0					0.166667	
	4				1					1
	6		1					0.857143		
	3		0.5					0.5		
	1				0					0.25
	4	1					1			
	3					1				
	3					1				
	7			1					1	
	3					1				
	4	1					1			

	4	1					1			
	2	1					0.666667			
	2									
ns										
	4				1					1
	0	0					0			
	7			1					1	
	3					1				
	4	1					1			
	2	0.5					0.5			
	3			1					0.6	
	3			0.5					0.5	
	4	1					1			
	6			1					1	
	8		1					1		
	4			1					0.571429	
	4	1					1			
	4									
	3					1				
	2			1					1	
	3					1				
	4				1					1
	3					1				
	4	1					1			
	1	0					0.25			
	3					1				
	7		1					1		
	5		1					0.833333		
	3			1					1	
	1		1					1		
	4	1					1			
	4				1					1
	7			1					1	
	3					1				
	4	1					1			
	6			1					1	
	2	1					1			
	3					1				
	0	0					0			
	0				0					0
	5			1					1	
	5			1					1	
ns										
	4									
	7		1					1		
	4	1					1			
	1		1					1		
	4				1					1
	6		1					0.857143		
	3					1				
	4	1					1			
	3			1					0.6	

	4				1					1
	3					1				
	4	1					1			
	6			1					1	
	4	1					1			
	3				1					1
	2	1					1			
	4									
	3					1				
	1			0					0.25	
	2			1					1	
	3					1				
	3					1				
	4	1					1			
	0	0					0			
	4				1					1
	0				0					0
	1	0					0.25			
	7		1					1		
	4	1					1			
	0					0				
	3			1					1	
	1		1					1		
	2									
	5		1					1		
	3					1				
	4			1					1	
	4				1					1
	1			0					0.166667	
	4				1					1
	6		1					0.857143		
	1				0					0.25
	3					1				
	4	1					1			
	4	1					1			
ns										
	7			1					1	
	2	0.5					0.5			
	3			0.5					0.5	
	6			1					1	
	8		1					1		
	4			1					0.571429	
	2			1					1	
	3					1				
	4				1					1
	3					1				
	3					1				
	4	1					1			
	4	1					1			
	6			1					1	
	2	1					1			
	0	0					0			

	0				0					0
	5			1					1	
	3					1				
	1			0					0.25	
	2			1					1	
	5			1					0.833333	
	4	1					1			
	1	0					0.25			
ns										
	3					1				
	3					1				
	4				1					1
	6		1					0.857143		
	3		0.5					0.5		
	4	1					1			
	7			1					1	
	3					1				
	4	1					1			
	2									
	4				1					1
	0	0					0			
	7			1					1	
	3			0.5					0.5	
	6			1					1	
	4	1					1			
	4									
	2			1					1	
	7		1					1		
	3			1					1	
	4	1					1			
	4				1					1
	4	1					1			
	6			1					1	
	3					1				
	1		1					1		
	4				1					1
	2	1					1			
	3					1				
	2			1					1	
	4				1					1
	3			1					1	
	2									
	4			1					1	
	4				1					1
	6		1					0.857143		
	4	1					1			
ns										
	7			1					1	
	2	0.5					0.5			
	4			1					0.571429	
	4				1					1
	3					1				

	3					1				
	4	1					1			
	4				1					1
	4	1					1			
	0					0				
	4	1					1			
	4			1					1	
	3					1				
	7			1					1	
	4	1					1			
ns										
	4	1					1			
	3					1				
	3					1				
	4				1					1
	3					1				
	4	1					1			
	7		1					1		
	4				1					1
	7			1					1	
	3					1				
	5			1					1	
	4	1					1			
	6		1					0.857143		
	4				1					1
	4	1					1			
	3				1					1
	4				1					1
	3			1					1	
	2									
	3					1				
	4				1					1
	4				1					1
	6		1					0.857143		
	3					1				
	6			1					1	
	2	1					1			
	0	0					0			
	2			1					1	
	5			1					0.833333	
	4	1					1			
	1	0					0.25			
	3					1				
	4				1					1
	2									
	4				1					1
	0	0					0			
	3			0.5					0.5	
	3					1				
	4			1					1	
	3					1				
	4				1					1

	0				0					0
ns										
	3					1				
	2	1					0.666667			
	6			1					1	
	4									
	2			1					1	
	3			1					1	
	3					1				
	6			1					1	
	1		1					1		
	3					1				
	4				1					1
	4	1					1			
	2	1					1			
	0	0					0			
	4				1					1
	1	0					0.25			
	0					0				
	4				1					1
	3			0.5					0.5	
	3					1				
	4	1					1			
ns										
	3					1				
	4				1					1
	4	1					1			
	3			0.5					0.5	
	6			1					1	
	4									
	7		1					1		
	4	1					1			
	4				1					1
	2			1					1	
	2									
	6		1					0.857143		
	7			1					1	
	7			1					1	
	4	1					1			
	3					1				
	4	1					1			
	7		1					1		
	4				1					1
	7			1					1	
	3					1				
	2									
	4	1					1			
	1	0					0.25			
	4	1					1			
	4									
	3					1				
	3					1				

	4	1					1			
	0	0					0			
	5			1					1	
	1	0					0.25			
	3					1				
ns										
	7		1					1		
	0					0				
	5		1					0.833333		
	1		1					1		
	4				1					1
	6		1					0.857143		
	1				0					0.25
	4	1					1			
	2									
ns										
	0	0					0			
	7			1					1	
	3					1				
	3			1					0.6	
	8		1					1		
	4									
	2			1					1	
	3					1				
	1	0					0.25			
	3					1				
	7		1					1		
	7			1					1	
	3					1				
	6			1					1	
	2	1					1			
	5			1					1	
	5			1					1	
ns										
	6		1					0.857143		
	4	1					1			
	4				1					1
	4	1					1			
	3				1					1
	2	1					1			
	4									
	3					1				
	1			0					0.25	
	3					1				
	3					1				
	0	0					0			
	1	0					0.25			
	4				1					1
	1				0					0.25
	3					1				
	4	1					1			
ns										

3			0.5					0.5	
6			1					1	
3					1				
4				1					1
0				0					0
5			1					1	
1			0					0.25	
2			1					1	
4	1					1			
3					1				
6		1					0.857143		
2									
3			0.5					0.5	
6			1					1	
3			1					1	
4				1					1
3					1				
4				1					1
2	1					1			
2			1					1	
4				1					1
4			1					0.571429	
3					1				
4			1					1	
7			1					1	
4				1					1
2									
2	1					1			
1	0					0.25			
2									
3			0.5					0.5	
4			1					1	
3					1				
4				1					1
0				0					0
3			1					1	
2	1					1			
0	0					0			
4				1					1
3					1				
4				1					1
4				1					1
2			1					1	
7			1					1	
4	1					1			
4				1					1
3					1				
4	1					1			
4	1					1			
6			1					1	
3			1					1	
4	1					1			

	0	yes	yes	CS	male
	0	no	yes	SVD	female
	0	no	yes	CS	female
	0	no	yes	SVD	female
1	0	yes	yes	SVD	male
1	0	no	yes	SVD	male
	1	yes	no	ISt	male
	0	no	no	ISt	female
	0	yes	yes	SVD	female
1	0	no	yes	SVD	male
	0	no	yes	SVD	male
1	0	yes	yes	SVD	female
	0	no	no	ISt	male
	0	no	yes	CS	female
	1	yes	no	ISt	female
	0	no	no	ISt	female
	1	yes	no	ISt	female
	0	no	yes	SVD	male
	0	no	yes	ISt	male
	1	yes	yes	SVD	male
1	0	yes	yes	SVD	female
	0	yes	no	CS	female
	0	yes	yes	ISt	male
	0	yes	yes	SVD	female
	0	yes	yes	SVD	female
1	0	yes	yes	SVD	female
	0	yes	yes	SVD	male
0	1	yes	yes	SVD	male
	0	yes	no	CS	male
	0	yes	no	SVD	male
	0	yes	yes	SVD	female
	0	yes	yes	SVD	female
	0	no	no	SVD	female
	0	yes	yes	CS	male
	0	no	yes	SVD	male
1	0	yes	yes	CS	male
1	0	no	yes	SVD	female
	0	no	no	ISt	female
	0	no	yes	SVD	male
	0	yes	yes	SVD	female
	0	no	yes	SVD	female
	1	yes	no	CS	female
	0	yes	no	ISt	female
	0	no	no	SVD	female
	1	yes	yes	SVD	male
	0	yes	yes	ISt	female
	0	yes	yes	CS	male
1	0	yes	no	CS	male
1	0	no	yes	SVD	male
	0	yes	yes	SVD	male
1	0	yes	no	SVD	female
	0	no	no	SVD	male

	0	yes	yes	SVD	female
	1	yes	yes	ISt	male
	0	yes	yes	ISt	male
	0	yes	no	CS	female
	0	no	no	SVD	male
	1	no	yes	SVD	female
	0	no	yes	CS	female
1	0	yes	no	SVD	female
	0	no	yes	SVD	male
	1	yes	yes	CS	male
	0	yes	yes	SVD	female
	0	no	yes	SVD	female
	0	no	no	SVD	male
	1	no	yes	SVD	female
	0	no	yes	SVD	male
	0	no	no	CS	female
	0	yes	yes	CS	male
	0	no	yes	SVD	female
1	0	yes	yes	SVD	male
	1	yes	yes	SVD	female
1	0	no	yes	SVD	male
	0	no	yes	SVD	male
1	0	yes	yes	SVD	female
	0	no	yes	CS	female
	1	yes	yes	SVD	male
1	0	yes	yes	SVD	female
	0	yes	yes	SVD	female
	0	yes	no	CS	male
	0	yes	yes	SVD	female
	0	yes	yes	SVD	female
	0	yes	yes	CS	male
	0	yes	no	ISt	female
	0	yes	yes	SVD	male
1	0	yes	no	SVD	female
	0	no	no	SVD	male
	0	no	yes	SVD	female
	0	no	yes	CS	female
1	0	yes	yes	SVD	male
	1	yes	no	ISt	female
	1	yes	no	ISt	female
	0	no	yes	SVD	male
	0	no	yes	ISt	male
	0	yes	no	CS	female
	0	yes	yes	ISt	male
	0	yes	yes	SVD	female
	0	yes	yes	SVD	female
	0	yes	yes	SVD	female
	0	yes	no	ISt	female
	0	no	no	SVD	female
1	0	yes	no	SVD	female
	0	yes	yes	SVD	female
	0	yes	yes	SVD	female

	0	no	yes	SVD	male
1	0	yes	yes	SVD	female
	1	no	no	SVD	male
	0	no	yes	SVD	female
	0	yes	yes	CS	male
	0	no	yes	SVD	female
	0	no	yes	CS	female
	0	no	yes	SVD	female
1	0	yes	yes	SVD	male
	1	yes	no	ISt	male
	0	yes	yes	SVD	female
1	0	no	yes	SVD	male
1	0	yes	yes	SVD	female
	0	no	yes	CS	female
	1	yes	no	ISt	female
	0	no	no	ISt	female
	1	yes	no	ISt	female
	1	yes	yes	SVD	male
	0	yes	yes	SVD	female
	0	yes	yes	SVD	female
0	1	yes	yes	SVD	male
	0	yes	yes	SVD	female
	0	yes	yes	SVD	female
	0	no	no	SVD	female
	0	no	yes	SVD	male
1	0	no	yes	SVD	female
	0	no	yes	SVD	male
	0	no	yes	SVD	female
	1	yes	no	CS	female
	0	yes	no	ISt	female
	0	no	no	SVD	female
	0	yes	yes	ISt	female
1	0	yes	no	CS	male
	0	no	no	SVD	male
	0	yes	yes	SVD	female
	0	yes	no	CS	female
	0	no	yes	CS	female
	1	yes	yes	CS	male
	0	no	yes	SVD	female
	0	no	yes	SVD	female
	0	no	yes	SVD	male
	0	no	no	CS	female
	0	yes	yes	SVD	female
1	0	no	yes	SVD	male
	0	no	yes	SVD	male
1	0	yes	yes	SVD	female
1	0	yes	yes	SVD	female
	1	yes	yes	CS	male
	0	no	no	SVD	male
	0	no	yes	SVD	female
	0	no	yes	CS	female
	1	yes	no	ISt	female

	1	yes	no	ISt	female
	0	no	yes	SVD	male
1	0	yes	yes	SVD	male
	1	yes	no	ISt	male
	0	yes	yes	SVD	female
	0	no	no	ISt	male
	0	no	yes	CS	female
	1	yes	yes	SVD	male
	0	yes	no	CS	female
1	0	yes	yes	SVD	female
1	0	yes	yes	CS	male
	0	yes	no	ISt	female
	0	no	no	SVD	female
	1	yes	yes	SVD	male
	0	yes	yes	CS	male
	0	yes	yes	SVD	male
1	0	yes	no	SVD	female
	0	yes	yes	SVD	female
	1	yes	yes	ISt	male
	0	no	no	SVD	male
	1	no	yes	SVD	female
	0	no	yes	CS	female
	0	no	yes	SVD	female
	0	no	yes	SVD	female
	0	yes	yes	CS	male
	0	no	yes	SVD	female
	0	yes	yes	SVD	female
	0	yes	yes	SVD	female
	0	yes	yes	SVD	female
	0	yes	yes	CS	male
	0	yes	no	ISt	female
	0	no	no	SVD	male
	0	no	yes	SVD	female
1	0	yes	yes	SVD	male
	0	yes	yes	SVD	female
	0	no	yes	SVD	male
	0	no	yes	CS	female
1	1	yes	yes	SVD	male
	0	yes	yes	SVD	female
	0	no	no	ISt	female
	0	yes	yes	SVD	female
	0	no	no	SVD	female
	0	no	yes	SVD	male
	0	yes	no	ISt	female
	0	no	no	SVD	female
	0	no	no	SVD	male
	0	yes	no	CS	female
	0	no	yes	CS	female
	1	yes	yes	CS	male
	0	no	no	CS	female
	0	no	yes	SVD	male
1	0	yes	yes	SVD	female

1	0	yes	yes	SVD	male
	0	no	yes	CS	female
	0	no	no	ISt	female
	0	yes	yes	SVD	female
0	1	yes	yes	SVD	male
	0	yes	yes	CS	male
	0	no	yes	SVD	male
1	0	yes	no	CS	male
	0	yes	yes	SVD	male
	0	no	no	SVD	male
	0	yes	no	CS	female
	0	no	no	SVD	male
1	0	yes	yes	SVD	male
1	0	no	yes	SVD	male
	0	no	yes	SVD	male
1	0	yes	yes	SVD	female
	0	no	yes	CS	female
	0	yes	yes	SVD	female
	0	yes	no	ISt	female
	0	yes	yes	SVD	male
1	0	yes	no	SVD	female
	0	no	yes	SVD	male
	0	yes	yes	SVD	female
	0	no	no	SVD	female
	0	no	yes	SVD	male
	0	no	no	SVD	male
	0	no	yes	SVD	female
	0	no	no	ISt	female
	0	yes	yes	SVD	female
	0	no	no	SVD	female
1	0	no	yes	SVD	female
	1	no	yes	SVD	female
	0	yes	no	ISt	female
	0	no	no	SVD	female
1	0	yes	no	CS	male
	0	no	yes	SVD	female
	0	no	yes	CS	female
	1	yes	no	ISt	female
	0	yes	yes	SVD	female
	0	no	no	ISt	male
	0	no	yes	CS	female
	1	yes	yes	SVD	male
1	0	yes	yes	SVD	female
	0	yes	no	ISt	female
	0	yes	yes	ISt	male
	0	no	no	SVD	male
	1	no	yes	SVD	female
	0	no	yes	SVD	female
1	0	yes	yes	SVD	male
	0	no	yes	SVD	male
1	0	no	yes	SVD	male
	0	no	no	ISt	female

	1	yes	no	ISt	female
	1	yes	no	CS	female
1	0	yes	yes	CS	male
	1	yes	yes	ISt	male
	0	no	yes	SVD	female
	0	no	yes	SVD	female
	0	yes	yes	SVD	female
	0	yes	yes	SVD	female
1	0	yes	no	SVD	female
	1	no	yes	SVD	female
	0	yes	yes	SVD	female
1	0	yes	no	SVD	female
	0	no	yes	SVD	male
	0	no	no	SVD	male
	0	no	yes	CS	female
	1	yes	no	ISt	female
	0	no	no	ISt	female
	1	yes	yes	SVD	male
0	1	yes	yes	SVD	male
	0	yes	no	ISt	female
	0	no	yes	SVD	female
1	0	yes	yes	SVD	female
	0	no	yes	CS	female
	0	yes	no	CS	female
1	0	yes	yes	CS	male
	0	yes	no	ISt	female
	0	yes	yes	SVD	female
	0	no	yes	SVD	female
	0	no	yes	SVD	female
	0	no	yes	SVD	female
	0	yes	yes	SVD	female
	0	yes	yes	CS	male
	0	yes	no	ISt	female
	0	yes	yes	SVD	female
	0	no	no	SVD	female
	1	no	no	SVD	female
	0	no	yes	CS	female
	0	yes	yes	SVD	male
	0	no	no	SVD	male
1	0	yes	yes	SVD	male
	0	no	yes	CS	female
	0	yes	yes	SVD	female
	0	yes	no	ISt	female
	0	yes	yes	SVD	male
1	0	yes	no	SVD	female
	0	no	no	SVD	female
	0	no	yes	CS	female
	1	yes	yes	SVD	male
	0	yes	yes	CS	male
	0	no	yes	SVD	female
1	0	yes	yes	SVD	male
1	0	yes	yes	SVD	female

	0	no	yes	CS	female
	1	yes	no	ISt	female
	0	no	yes	SVD	male
	1	yes	yes	SVD	male
1	0	yes	yes	SVD	female
	0	yes	no	CS	female
	0	yes	yes	SVD	female
0	1	yes	yes	SVD	male
	0	yes	no	CS	male
	0	yes	yes	SVD	female
	0	no	yes	SVD	female
	0	no	no	SVD	female
	0	yes	yes	ISt	female
	0	yes	yes	CS	male
	0	yes	yes	ISt	male
	0	yes	no	CS	female
	1	no	yes	SVD	female
	1	no	yes	CS	female
1	0	yes	no	SVD	female
	0	yes	yes	SVD	female
	0	no	yes	SVD	male
	0	no	yes	SVD	female
	0	yes	yes	SVD	female
1	0	no	yes	SVD	male
	1	yes	yes	SVD	male
1	0	yes	yes	SVD	female
	0	yes	yes	SVD	female
	0	yes	yes	SVD	male
1	0	yes	no	SVD	female
	0	no	yes	SVD	female
	0	no	yes	CS	female
	0	no	yes	SVD	male
	0	no	yes	ISt	male
	0	yes	no	CS	female
	0	no	no	SVD	female
	0	yes	yes	SVD	female
	0	no	yes	SVD	male
	0	no	no	SVD	male
	1	no	yes	SVD	female
	0	no	yes	CS	female
	0	no	yes	SVD	female
1	0	yes	yes	SVD	male
	1	yes	no	ISt	male
1	0	no	yes	SVD	male
1	0	yes	yes	SVD	female
	1	yes	no	ISt	female
	1	yes	yes	SVD	male
	0	no	yes	SVD	female
	0	yes	yes	ISt	female
1	1	yes	no	CS	male
	0	yes	yes	SVD	female
	0	yes	no	CS	female

	0	no	yes	SVD	female
	0	no	yes	SVD	female
1	0	no	yes	SVD	male
	0	no	yes	SVD	male
	1	yes	no	ISt	female
	0	no	yes	SVD	male
	1	yes	no	ISt	male
	1	yes	yes	SVD	female
	0	no	yes	CS	female
1	0	yes	yes	CS	male
	0	no	no	SVD	female
	0	yes	yes	ISt	male
	0	no	yes	SVD	female
	0	no	yes	SVD	female
	0	yes	yes	SVD	female
	1	yes	no	ISt	female
1	0	yes	yes	SVD	male
	0	no	yes	SVD	male
	0	no	yes	CS	female
	0	yes	yes	SVD	female
	0	yes	no	ISt	female
	0	no	no	CS	female
1	0	yes	yes	SVD	male
	1	no	yes	SVD	male
	0	yes	yes	SVD	male
	0	no	yes	SVD	male
	0	no	no	SVD	female
	0	no	yes	CS	female
	1	yes	yes	SVD	male
	0	yes	yes	ISt	male
	0	no	yes	SVD	female
	0	no	yes	SVD	male
1	0	no	yes	SVD	male
	0	no	no	ISt	female
	1	yes	no	ISt	female
	0	yes	yes	SVD	female
	0	no	yes	CS	female
	1	yes	no	ISt	female
	0	yes	no	ISt	female
1	1	yes	yes	CS	male
	0	yes	no	ISt	female
	0	yes	no	ISt	female
	0	yes	yes	SVD	female
	0	no	yes	CS	female
	0	no	no	SVD	male
	0	yes	no	ISt	female
1	0	yes	no	SVD	female
	0	no	yes	CS	female
	0	yes	yes	CS	male
	0	no	yes	SVD	female
	0	yes	yes	SVD	female
	1	yes	yes	CS	male

0	no	yes	SVD	female
